# Lower Parathyroid Hormone Levels are Associated With Reduced Fracture Risk in Japanese Patients on Hemodialysis

**DOI:** 10.1016/j.ekir.2024.07.008

**Published:** 2024-07-18

**Authors:** Hirotaka Komaba, Takahiro Imaizumi, Takayuki Hamano, Naohiko Fujii, Masanori Abe, Norio Hanafusa, Masafumi Fukagawa

**Affiliations:** 1 Committee of Renal Data Registry, Japanese Society for Dialysis Therapy, Tokyo, Japan; 2 Division of Nephrology, Endocrinology and Metabolism, Tokai University School of Medicine, Isehara, Japan; 3 The Institute of Medical Sciences, Tokai University, Isehara, Japan; 4 Department of Advanced Medicine, Nagoya University Hospital, Nagoya, Japan; 5 Department of Nephrology, Nagoya University Graduate School of Medicine, Nagoya, Japan; 6 Department of Nephrology, Nagoya City University Graduate School of Medical Sciences, Nagoya, Japan; 7 Department of Nephrology, Osaka University Graduate School of Medicine, Suita, Japan; 8 Department of Nephrology, Hyogo Prefectural Nishinomiya Hospital, Nishinomiya, Japan; 9 Division of Nephrology, Hypertension and Endocrinology, Department of Internal Medicine, Nihon University School of Medicine, Tokyo, Japan; 10 Department of Blood Purification, Tokyo Women’s Medical University, Tokyo, Japan

**Keywords:** fracture, hemodialysis, parathyroid hormone, secondary hyperparathyroidism

## Abstract

**Introduction:**

Secondary hyperparathyroidism (SHPT) affects bone metabolism and may lead to bone fragility. However, there is conflicting evidence as to whether parathyroid hormone (PTH) levels are associated with fracture risk and whether the relationship is linear or U-shaped.

**Methods:**

We examined the association between PTH levels and the risk of any fracture and site-specific fractures in a nationwide cohort of 180,333 patients on hemodialysis. We also examined the association between the percent change in PTH levels during the preceding 1 year and subsequent fracture.

**Results:**

At baseline, the median intact PTH level was 141 pg/ml (interquartile range, 78–226 pg/ml). During 1 year of follow-up, there were a total of 3762 fractures requiring hospitalization (1361 hip, 551 vertebral, and 1850 other). In an adjusted analysis, higher baseline PTH levels were associated with an incrementally increased risk of any fracture (odds ratio [OR] per doubling of intact PTH, 1.06; 95% confidence interval, 1.03–1.09). The association between PTH levels and fracture risk was more pronounced for hip fractures but not found for vertebral fractures. The absolute risk difference associated with higher PTH levels appeared to be more pronounced in older individuals, females, and those with lower body mass index (BMI). Change in PTH levels was also associated with fracture risk: the adjusted OR for fracture decreased linearly with decreasing PTH levels over 1 year, regardless of the preceding PTH levels.

**Conclusion:**

Lower PTH levels are associated with a graded reduction in fracture risk. Further studies are needed to determine whether intensive PTH control reduces fracture risk.

## Graphical abstract

**Figure:**
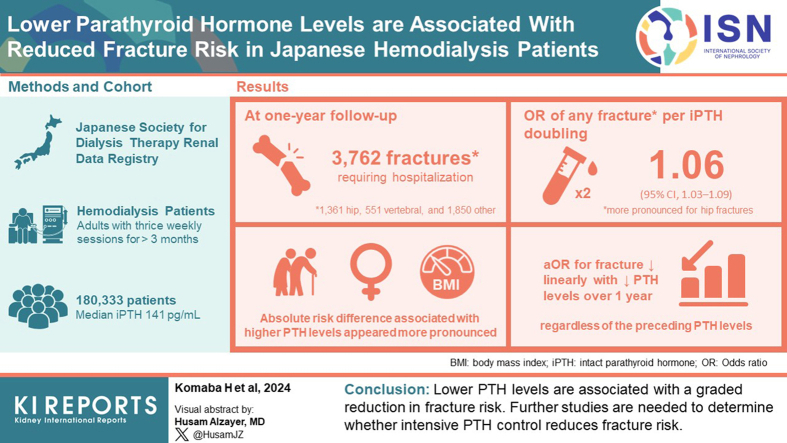

See Commentary on Page 2854

SHPT is a common complication in patients undergoing hemodialysis, with approximately 70% to 80% of patients worldwide receiving medication for this condition.^1^^,^^2^ Although the target ranges for PTH in current clinical guidelines are mainly based on its associations with mortality,^3^^,^^4^ the primary target organ of PTH is bone in the setting of kidney failure. Thus, bone disease has long been the most recognized consequence of SHPT.^5^ Persistently elevated PTH leads to high-turnover bone disease, characterized by excessive bone resorption and formation in trabecular bone;^5^ however, it also induces deterioration of cortical bone architecture,^6^, ^7^, ^8^ which has a more pronounced impact on bone strength.^9^^,^^10^ Conversely, over suppression of PTH can lead to low bone turnover, typically referred to as adynamic bone disease.^11^ This condition has been speculated to cause bone fragility; however, previous bone biopsy series have not supported this possibility.^12^^,^^13^

To date, several observational studies have examined the relationship between PTH levels and fracture risk in patients on dialysis, with mixed results. Consistent with the deleterious effects of elevated PTH on bone, large studies have found a linear increase in risk with higher PTH levels^14^ or an increased risk with very high PTH levels.^15^^,^^16^ However, other studies have found no association,^17^^,^^18^ a U-shaped association with increased risk at both ends,^19^ or an increased risk at low PTH levels;^20^^,^^21^ the latter 2 of which may suggest increased bone fragility at low PTH levels. These inconsistent results may be attributed to small sample sizes in some studies, varying degrees of adjustment for confounders, and differences in the type of fracture outcomes (any vs. site-specific fracture). Given the reported increase in adynamic bone disease in recent years^22^ and the introduction of several types of calcimimetics that provide adequate PTH suppression,^23^, ^24^, ^25^ it is increasingly important to determine whether and how very low PTH levels are associated with fracture risk.

The purpose of this study was to examine the risk of any fracture and site-specific fractures across a range of PTH levels, using prospective data from a nationwide registry of patients on dialysis in Japan. In addition, we examined the association between 1-year or 2-year percent change in PTH levels and subsequent fracture risk.

## Methods

### Data Source

This study used data from the Japanese Society for Dialysis Therapy (JSDT) Renal Data Registry, a nationwide database of patients on dialysis in Japan. Details of the database have been described elsewhere.^26^^,^^27^ Briefly, the JSDT sends questionnaires to all dialysis facilities in Japan at the end of each year to prospectively collect data on demographics, comorbid conditions, laboratory values, prescriptions, and clinical outcomes. These data are collected by medical staff at each facility using uniform and standardized data collection tools. The database covers nearly all patients on dialysis in Japan: the response rate to the questionnaire was 98.6% in 2016^26^ and 98.8% in 2017.^27^ In addition to the routine questionnaire, the JSDT collected data on hospitalization events for the first time in its history in 2017.^27^ The study protocol was approved by the Medicine Ethics Committee of the JSDT, which waived the need for informed consent because the data were anonymized before being transferred to the investigators.

### Study Population

Patients were eligible for inclusion in the study if they were aged 18 years or older, were receiving maintenance hemodialysis or hemodiafiltration thrice weekly for more than 3 months, had data on serum albumin, calcium, phosphorus, and intact or whole PTH at the end of 2016, and had hospitalization data available in 2017.

### Baseline Covariates

The following information was obtained from the database at the end of 2016: age, sex, dialysis duration, cause of kidney failure, dialysis modality, height, postdialysis weight, single-pool Kt/V, normalized protein catabolic rate, history of cardiovascular disease (myocardial infarction, cerebral infarction, cerebral hemorrhage, and amputation), history of hip fracture, hemoglobin, albumin, creatinine, calcium, phosphorus, PTH (intact or whole), total cholesterol, and C-reactive protein. We also obtained biochemical parameters at the end of 2014 and 2015 to calculate the percent change in PTH levels during the preceding 1 or 2 years and subsequent fracture risk (see below). Data on prescriptions (e.g., phosphate binders, active vitamin D, and calcimimetics) and history of parathyroidectomy were not collected in 2016 and were therefore not included in the analysis. In the study cohort, PTH levels were measured using the whole PTH assay in 8.4% of patients. These values were multiplied by 1.7 to obtain an equivalent intact PTH value.^4^ Serum calcium levels were corrected for the albumin concentration using Payne’s formula.^28^

### Exposures and Outcomes

**Figure 1:**
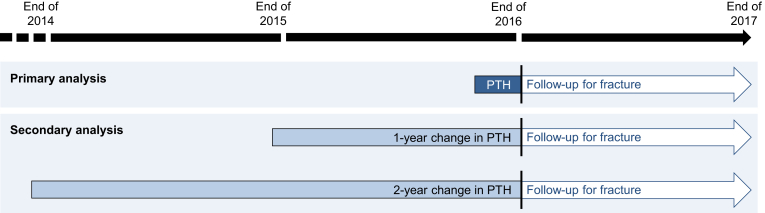
Schematic illustration of the study design. The primary analysis examined the association between PTH levels at the end of 2016 and fracture events during 2017, adjusted for covariates at the end of 2016. Secondary analyses examined the association between 1-year and 2-year change in PTH levels between 2015 and 2016 and between 2014 and 2016, respectively, and fracture events during 2017. For adjustment, we used covariates at the time of the first PTH measurement (e.g., in the 1-year change analysis, covariates at the end of 2015 were used for adjustment). PTH, parathyroid hormone.

The primary exposure was intact PTH levels at the end of 2016. Secondary exposures included^1^ 1-year and 2-year percent change in intact PTH levels between 2015 and 2016 and between 2014 and 2016, respectively, and^2^ serum calcium and phosphorus levels at the end of 2016. The primary outcome was a composite of hospitalization for hip, vertebral, and other fractures during 2017. Secondary outcomes were hospitalization for site-specific fractures. The study schema is illustrated in Figure 1.

### Statistical Analysis

Standard descriptive statistics were used to compare the demographic and clinical characteristics of the study population according to intact PTH, calcium, and phosphorus. We used logistic regression analysis to estimate ORs for overall and site-specific fractures associated with intact PTH, calcium, and phosphorus. We examined these biochemical parameters both as continuous variables and categorized them into deciles. Because the distribution of intact PTH was highly skewed, we evaluated intact PTH as a continuous variable after log2 transformation, interpreted as “per doubling.” In the categorical model for intact PTH, the lowest decile was defined as the reference because this group had the lowest fracture risk in the preliminary analysis. For the analysis of serum calcium and phosphorus, the fourth decile was defined *a priori* as the reference. To examine potential nonlinear associations between each exposure of interest and fracture, we used restricted cubic spline (RCS) analysis based on adjusted logistic regression models with 3 knots at the 10th, 50th, and 90th percentiles.^29^ In this continuous model, the reference values of intact PTH 60 pg/ml, calcium 8.4 mg/dl, and phosphorus 3.5 mg/dl were selected *a priori* according to the lower limit of the target ranges recommended by the JSDT guideline.^4^ We also estimated the probability of fracture at different levels of intact PTH using the RCS analysis.

We estimated ORs and 95% confidence intervals for fracture adjusted for incremental sets of potential confounders. Model 1 adjusted for age, sex, dialysis duration, cause of kidney failure, dialysis modality (hemodialysis or hemodiafiltration), BMI, Kt/V, normalized protein catabolic rate, history of cardiovascular disease (myocardial infarction, cerebral infarction, cerebral hemorrhage, and amputation), and history of hip fracture. Model 2 further adjusted for hemoglobin, albumin, creatinine, total cholesterol, C-reactive protein, and the 2 other predictor variables (e.g., in the PTH model, calcium and phosphorus were included as adjusting covariates).

We examined the adjusted ORs for fracture associated with intact PTH in subgroups of age, sex, dialysis duration, diabetes (as the cause of kidney failure), BMI, prior hip fracture, albumin, and creatinine, and tested for the interaction of each of these variables with intact PTH. To further investigate whether the absolute difference in fracture rates between different levels of intact PTH could be modified by patient characteristics, we estimated the probability of fracture across intact PTH levels using RCS analysis in subgroups of age, sex, and BMI.

In addition to the single point measures of intact PTH, we calculated the percent change in intact PTH during the preceding 1 and 2 years and examined their associations with subsequent fracture, expressed as ORs per 30% reduction in intact PTH. We also examined these associations by RCS analysis. For multivariable adjustment, we used covariates at the time of the first intact PTH measurement (e.g., in the analysis of the 1-year change between 2015 and 2016, covariates in 2015 were used for adjustment). We examined whether the adjusted ORs per 30% reduction in intact PTH could be modified by the baseline intact PTH (e.g., in the analysis of the 1-year change between 2015 and 2016, intact PTH in 2015 was used as the baseline). We also examined the absolute risk reduction and bootstrapped 95% confidence intervals^30^ for any fracture and hip fracture associated with >30% reduction in intact PTH over 1 year, and estimated the number-needed-to-be-exposed^31^ to >30% reduction in intact PTH to prevent 1 any fracture and 1 hip fracture over 1 year in the overall population and in subgroups of age, sex, and BMI.

We performed several sensitivity analyses to test the robustness of our findings as follows: (i) a Poisson regression analysis to model the number of all fractures; (ii) a complete case analysis that excluded all individuals with missing data; (iii) an analysis using the average of 2 (i.e., 2015 and 2016) or 3 (i.e., 2014, 2015, and 2016) annual measurements of mineral metabolism parameters; (iv) a multinomial logistic regression analysis to account for death as a competing event; and (v) an analysis that separately examined patients whose PTH levels were measured by the intact PTH assay and those measured by the whole PTH assay.

The proportion of missing data was <15% for all baseline variables (Supplementary Table S1). We imputed missing data by using multiple imputation to create 10 imputed data sets. Statistical analyses were performed on each imputed dataset and then pooled to obtain single parameter estimates. Two-sided *P* values of <0.05 were considered statistically significant. Statistical analyses were performed using IBM SPSS Statistics 24 (IBM) and Stata version 18.0 (StataCorp).

## Results

### Study Population

**Figure 2:**
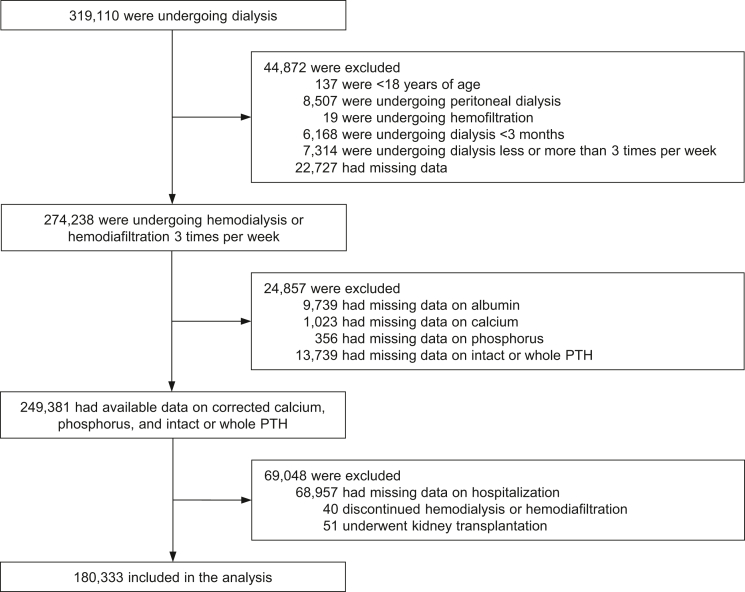
Study profile. PTH, parathyroid hormone.

We identified 180,333 patients on hemodialysis who met our inclusion criteria (Figure 2). The baseline characteristics are shown in Table 1 for the overall population and by deciles of intact PTH. The median intact PTH level was 141 pg/ml (interquartile range, 78–226 pg/ml), the mean ± SD serum calcium level was 9.1 ± 0.7 mg/dl, the mean ± SD serum phosphorus level was 5.2 ± 1.4 mg/dl, and 4.5% of participants had a history of hip fracture. Patients with higher intact PTH levels tended to be younger; were less likely to have diabetes as the cause of kidney failure; and had longer dialysis duration, higher BMI, higher creatinine, lower calcium, and higher phosphorus levels.

**Table 1:** Baseline characteristics by deciles of intact PTH

Characteristic	Overall	Deciles of intact PTH									
		Decile 1	Decile 2	Decile 3	Decile 4	Decile 5	Decile 6	Decile 7	Decile 8	Decile 9	Decile 10
		<39 pg/ml	39–65 pg/ml	66–90 pg/ml	91–116 pg/ml	117–141 pg/ml	142–170 pg/ml	171–204 pg/ml	205–252 pg/ml	253–337 pg/ml	>337 pg/ml
	(*n* = 180,333)	(*n* = 18,473)	(*n* = 17,847)	(*n* = 17,812)	(*n* = 18,088)	(*n* = 18,183)	(*n* = 18,301)	(*n* = 17,611)	(*n* = 17,964)	(*n* = 18,046)	(*n* = 18,008)
Age, yr	67.6 ± 12.2	68.8 ± 11.8	68.7 ± 12.0	68.4 ± 12.0	68.4 ± 11.9	68.2 ± 11.9	67.9 ± 12.0	67.5 ± 12.2	67.0 ± 12.4	66.5 ± 12.5	64.7 ± 13.0
Female, %	35.8	39.3	36.7	36.0	35.3	34.6	34.2	33.8	34.3	34.9	38.7
Dialysis duration, mo	70 (32–139)	65 (30–143)	66 (31–131)	67 (32–132)	69 (32–134)	69 (32–135)	70 (32–138)	71 (32–137)	70 (31–137)	73 (32–140)	90 (38–159)
Cause of kidney failure, %											
Glomerulonephritis	29.7	30.7	28.3	28.4	28.9	29.1	28.8	29.5	29.5	30.1	33.9
Diabetes	38.5	39.2	39.9	39.7	39.2	39.1	39.2	39.6	38.4	37.7	32.7
Hypertension	9.8	8.7	9.9	9.9	10.1	9.9	10.1	9.6	10.1	10.1	9.2
Others	12.1	12.0	12.1	11.8	11.8	12.0	12.1	11.9	12.2	12.1	13.4
Unknown	9.9	9.4	9.8	10.2	10.0	10.0	9.8	9.4	9.8	10.1	10.9
Hemodiafiltration, %	25.1	23.6	24.4	25.0	25.7	25.7	25.4	25.7	24.5	25.2	26.0
Body mass index, kg/m^2^	21.9 ± 4.0	21.2 ± 3.7	21.5 ± 3.8	21.6 ± 3.9	21.8 ± 3.9	21.9 ± 4.0	22.0 ± 3.9	22.1 ± 4.0	22.2 ± 4.0	22.3 ± 4.2	22.4 ± 4.4
*Kt/V*	1.48 ± 0.31	1.50 ± 0.32	1.50 ± 0.32	1.49 ± 0.31	1.48 ± 0.31	1.48 ± 0.31	1.48 ± 0.31	1.47 ± 0.31	1.47 ± 0.31	1.46 ± 0.30	1.48 ± 0.31
nPCR, g/kg/d	0.85 ± 0.17	0.84 ± 0.18	0.84 ± 0.17	0.84 ± 0.17	0.84 ± 0.17	0.85 ± 0.17	0.85 ± 0.17	0.86 ± 0.17	0.86 ± 0.17	0.87 ± 0.17	0.89 ± 0.17
Past history, %											
Myocardial infarction	9.7	9.2	9.7	9.9	9.8	9.6	9.6	10.0	9.8	10.1	9.1
Cerebral infarction	17.1	17.8	18.8	17.7	17.4	17.3	17.6	16.5	16.3	16.7	15.4
Cerebral hemorrhage	6.2	6.9	6.9	6.1	6.3	6.1	5.8	5.8	5.9	5.8	6.2
Amputation	3.4	3.6	3.5	3.6	3.5	3.5	3.2	3.1	3.3	3.3	3.3
Hip fracture	4.5	5.3	5.3	4.4	4.7	4.3	4.5	3.9	4.0	4.1	4.2
Laboratory tests											
Hemoglobin, g/dl	10.9 ± 1.2	10.8 ± 1.3	10.8 ± 1.3	10.8 ± 1.2	10.9 ± 1.2	10.9 ± 1.2	10.9 ± 1.2	10.9 ± 1.2	10.9 ± 1.2	10.9 ± 1.2	10.9 ± 1.3
Albumin, g/dl	3.6 ± 0.4	3.6 ± 0.4	3.6 ± 0.4	3.6 ± 0.4	3.6 ± 0.4	3.6 ± 0.4	3.6 ± 0.4	3.6 ± 0.4	3.6 ± 0.4	3.6 ± 0.4	3.6 ± 0.4
Creatinine, mg/dl	10.1 ± 2.8	9.7 ± 2.7	9.8 ± 2.7	9.9 ± 2.8	10.0 ± 2.8	10.1 ± 2.7	10.2 ± 2.7	10.2 ± 2.7	10.3 ± 2.8	10.5 ± 2.8	10.7 ± 2.8
Calcium, mg/dl	9.1 ± 0.7	9.5 ± 0.8	9.3 ± 0.7	9.2 ± 0.7	9.2 ± 0.7	9.1 ± 0.7	9.1 ± 0.7	9.1 ± 0.7	9.0 ± 0.7	9.0 ± 0.7	9.0 ± 0.8
Phosphorus, mg/dl	5.2 ± 1.4	5.0 ± 1.4	5.0 ± 1.4	5.0 ± 1.3	5.1 ± 1.3	5.2 ± 1.3	5.2 ± 1.3	5.3 ± 1.3	5.4 ± 1.4	5.5 ± 1.4	5.8 ± 1.6
Intact PTH, pg/ml	141 (78–226)	23 (14–31)	53 (46–59)	78 (72–84)	103 (96–109)	128 (122–135)	155 (148–163)	187 (178–195)	226 (215–238)	286 (267–308)	443 (379–574)
Total cholesterol, mg/dl	157 ± 36	156 ± 36	156 ± 35	156 ± 36	156 ± 35	156 ± 35	157 ± 35	157 ± 35	158 ± 36	159 ± 35	162 ± 37
CRP, mg/dl	0.14 (0.05–0.40)	0.14 (0.06–0.44)	0.14 (0.06–0.44)	0.13 (0.05–0.40)	0.13 (0.05–0.40)	0.13 (0.05–0.40)	0.13 (0.05–0.39)	0.13 (0.05–0.39)	0.13 (0.05–0.38)	0.13 (0.05–0.38)	0.15 (0.06–0.41)

CRP, C-reactive protein; *Kt/V*, dialysis adequacy; nPCR, normalized protein catabolic rate; PTH, parathyroid hormone.

Data are percentage, mean ± SD, or median (interquartile range). Percentages do not add up to 100% in some cases because of rounding.

### Baseline PTH and Fracture Risk

During the 1-year follow-up, there were a total of 3762 fractures requiring hospitalization (1361 hip, 551 vertebral, and 1850 other), corresponding to an incidence rate of 21.1 per 1000 patient-years: 3708 participants experienced at least 1 fracture, and 54 experienced 2 fractures (3 with hip and vertebral, 37 with hip and other, and 14 with vertebral and other). A total of 3375 participants died during the follow-up.

**Figure 3:**
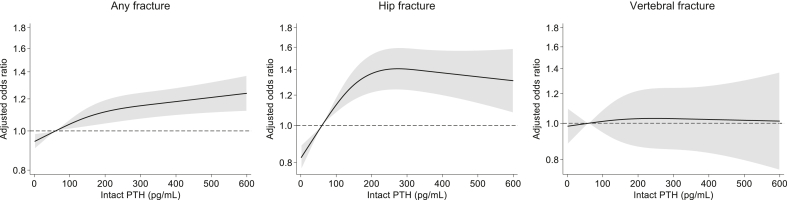
Restricted cubic splines of the adjusted odds ratio for any fracture (left), hip fracture (middle), and vertebral fracture (right) according to intact parathyroid hormone (PTH). Models adjusted for age, sex, dialysis duration, cause of kidney failure, dialysis modality (hemodialysis or hemodiafiltration), body mass index, Kt/V, normalized protein catabolic rate, history of cardiovascular disease (myocardial infarction, cerebral infarction, cerebral hemorrhage, and amputation), history of hip fracture, hemoglobin, albumin, creatinine, calcium, phosphorus, total cholesterol, and C-reactive protein. The black solid line represents the odds ratio, and the gray area represents the 95% confidence interval.

In Table 2, we show the unadjusted and multivariable-adjusted ORs for any fracture, hip fracture, and vertebral fracture according to intact PTH levels, expressed as a continuous variable and in deciles. In unadjusted models, there were no significant associations between intact PTH levels and fracture risk. However, after adjustment for covariates in model 1 or 2, there was a strong association between higher intact PTH levels and an incrementally increased risk of any fracture, with the lowest risk at the lowest decile of intact PTH <39 pg/ml. Higher intact PTH levels were also associated with an increased risk of hip fracture: the magnitude of the excess risk was greater than that for any fracture. By contrast, there was no association between intact PTH levels and vertebral fracture. An RCS model showed a relatively linear relationship between intact PTH levels and the risk of any fracture (Figure 3). The risk of hip fracture increased more steeply than any fracture with higher intact PTH levels, with an apparently monotonic increase in risk until intact PTH levels exceeded approximately 240 pg/ml.

**Table 2:** Logistic regression analysis for the association between intact PTH and fracture

Outcome	Intact PTH	Range	N patients	N events	OR (95% CI)		
					Unadjusted	Model 1	Model 2
Any fracture	Per doubling		180,333	3,708	1.00 (0.98–1.03)	1.06 (1.03–1.08)	1.06 (1.03–1.09)
	Decile 1	<39 pg/ml	18,473	365	Reference	Reference	Reference
	Decile 2	39–65 pg/ml	17,847	371	1.05 (0.91–1.22)	1.08 (0.93–1.25)	1.09 (0.94–1.27)
	Decile 3	66–90 pg/ml	17,812	381	1.08 (0.94–1.25)	1.16 (1.00–1.34)	1.18 (1.02–1.37)
	Decile 4	91–116 pg/ml	18,088	390	1.09 (0.95–1.26)	1.18 (1.02–1.37)	1.21 (1.04–1.40)
	Decile 5	117–141 pg/ml	18,183	388	1.08 (0.94–1.25)	1.20 (1.03–1.38)	1.22 (1.06–1.42)
	Decile 6	142–170 pg/ml	18,301	359	0.99 (0.86–1.15)	1.12 (0.96–1.30)	1.14 (0.98–1.33)
	Decile 7	171–204 pg/ml	17,611	364	1.05 (0.90–1.21)	1.22 (1.05–1.41)	1.24 (1.07–1.44)
	Decile 8	205–252 pg/ml	17,964	373	1.05 (0.91–1.22)	1.25 (1.08–1.44)	1.27 (1.09–1.48)
	Decile 9	253–337 pg/ml	18,046	364	1.02 (0.88–1.18)	1.23 (1.06–1.42)	1.25 (1.08–1.46)
	Decile 10	>337 pg/ml	18,008	353	0.99 (0.86–1.15)	1.25 (1.08–1.46)	1.26 (1.08–1.47)
Hip fracture	Per doubling		180,333	1,361	1.03 (0.99–1.07)	1.10 (1.06–1.15)	1.11 (1.06–1.16)
	Decile 1	<39 pg/ml	18,473	119	Reference	Reference	Reference
	Decile 2	39–65 pg/ml	17,847	139	1.21 (0.95–1.55)	1.27 (0.99–1.63)	1.30 (1.01–1.67)
	Decile 3	66–90 pg/ml	17,812	125	1.09 (0.85–1.40)	1.21 (0.94–1.56)	1.25 (0.97–1.62)
	Decile 4	91–116 pg/ml	18,088	136	1.17 (0.91–1.50)	1.33 (1.04–1.70)	1.38 (1.07–1.78)
	Decile 5	117–141 pg/ml	18,183	131	1.12 (0.87–1.44)	1.31 (1.02–1.68)	1.37 (1.06–1.76)
	Decile 6	142–170 pg/ml	18,301	148	1.26 (0.99–1.60)	1.52 (1.19–1.94)	1.59 (1.24–2.03)
	Decile 7	171–204 pg/ml	17,611	161	1.42 (1.12–1.81)	1.81 (1.42–2.30)	1.89 (1.48–2.41)
	Decile 8	205–252 pg/ml	17,964	140	1.21 (0.95–1.55)	1.57 (1.23–2.02)	1.65 (1.28–2.12)
	Decile 9	253–337 pg/ml	18,046	140	1.21 (0.94–1.54)	1.59 (1.24–2.04)	1.66 (1.29–2.14)
	Decile 10	>337 pg/ml	18,008	122	1.05 (0.82–1.36)	1.48 (1.15–1.92)	1.53 (1.18–1.98)
Vertebral fracture	Per doubling		180,333	551	0.95 (0.89–1.00)	1.00 (0.94–1.06)	1.01 (0.95–1.08)
	Decile 1	<39 pg/ml	18,473	65	Reference	Reference	Reference
	Decile 2	39–65 pg/ml	17,847	53	0.84 (0.59–1.21)	0.85 (0.60–1.18)	0.87 (0.62–1.23)
	Decile 3	66–90 pg/ml	17,812	60	0.96 (0.67–1.36)	1.00 (0.71–1.41)	1.06 (0.75–1.49)
	Decile 4	91–116 pg/ml	18,088	69	1.08 (0.77–1.52)	1.15 (0.97–1.37)	1.24 (0.92–1.66)
	Decile 5	117–141 pg/ml	18,183	56	0.87 (0.61–1.25)	0.95 (0.67–1.35)	1.03 (0.72–1.47)
	Decile 6	142–170 pg/ml	18,301	57	0.88 (0.62–1.26)	0.98 (0.82–1.18)	1.07 (0.77–1.51)
	Decile 7	171–204 pg/ml	17,611	46	0.74 (0.51–1.08)	0.85 (0.59–1.22)	0.93 (0.64–1.35)
	Decile 8	205–252 pg/ml	17,964	55	0.87 (0.61–1.25)	1.03 (0.86–1.24)	1.12 (0.80–1.57)
	Decile 9	253–337 pg/ml	18,046	47	0.74 (0.51–1.08)	0.90 (0.74–1.10)	0.98 (0.68–1.42)
	Decile 10	>337 pg/ml	18,008	43	0.68 (0.46–1.00)	0.90 (0.63–1.30)	0.95 (0.65–1.39)

CI, confidence interval; OR, odds ratio; PTH, parathyroid hormone.

Model 1 adjusted for age, sex, dialysis duration, cause of kidney failure, dialysis modality (hemodialysis or hemodiafiltration), body mass index, *Kt/V*, normalized protein catabolic rate, history of cardiovascular disease (myocardial infarction, cerebral infarction, cerebral hemorrhage, and amputation), and history of hip fracture.

Model 2 adjusted for Model 1 covariates plus hemoglobin, albumin, creatinine, calcium, phosphorus, total cholesterol, and C-reactive protein.

**Figure 4:**
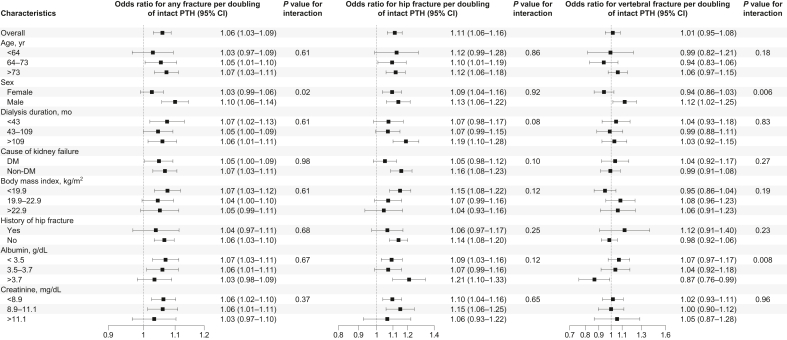
Subgroup analysis of fracture risk associated with intact parathyroid hormone (PTH). Models adjusted for age, sex, dialysis duration, cause of kidney failure, dialysis modality (hemodialysis or hemodiafiltration), body mass index, Kt/V, normalized protein catabolic rate, history of cardiovascular disease (myocardial infarction, cerebral infarction, cerebral hemorrhage, and amputation), history of hip fracture, hemoglobin, albumin, creatinine, calcium, phosphorus, total cholesterol, and C-reactive protein. Squares represent point estimates of the odds ratio, and horizontal lines indicate 95% confidence intervals (CIs). DM, diabetes mellitus.

**Figure 5:**
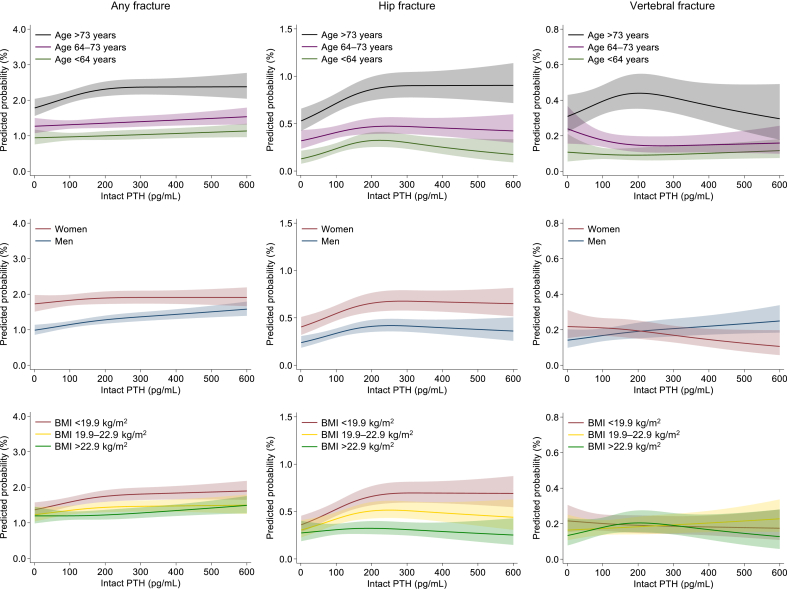
Predicted probability of any fracture (left), hip fracture (middle), and vertebral fracture (right) across intact parathyroid hormone (PTH) levels, stratified by age (upper), sex (middle), and body mass index (BMI). Models adjusted for age, sex, dialysis duration, cause of kidney failure, dialysis modality (hemodialysis or hemodiafiltration), BMI, Kt/V, normalized protein catabolic rate, history of cardiovascular disease (myocardial infarction, cerebral infarction, cerebral hemorrhage, and amputation), history of hip fracture, hemoglobin, albumin, creatinine, calcium, phosphorus, total cholesterol, and C-reactive protein. The dark solid line represents the predicted probability of fracture, and the light-colored area represents the 95% confidence interval.

In stratified analysis, the association between intact PTH levels and the risk of any fracture or hip fracture was largely consistent across most strata (Figure 4). For vertebral fracture, there was a significant effect modification by sex and albumin: the association between intact PTH levels and fracture risk was more pronounced in males and patients with higher albumin levels (*P* value for interaction = 0.006 and 0.008, respectively). The estimated probability of fracture across intact PTH levels is shown in Supplementary Figure S1 for the overall population, and in Figure 5 for subgroups of age, sex, and BMI. The estimated probability of fracture was higher in older individuals, females, and those with lower BMI; and the absolute risk difference associated with higher PTH levels appeared to be more pronounced in these subgroups.

In sensitivity analyses, the associations between intact PTH and fracture risk were qualitatively unchanged when we analyzed the number of all fractures using Poisson regression models (Supplementary Table S2); when we performed a complete case analysis (Supplementary Table S3); and when we substituted averages of annual intact PTH measurements over 2 or 3 years instead of single measurements (Supplementary Tables S4 and S5). Because higher intact PTH levels were also associated with an increased risk of death (Supplementary Figure S2 and Supplementary Table S6), we performed a multinomial logistic regression analysis to account for death as a competing event. The results of the multinomial logistic regression analysis were consistent with the primary results (Supplementary Table S7). As an additional sensitivity analysis, we examined whether the type of PTH assay (i.e., intact or whole) modified the association between PTH levels and fracture risk. Baseline characteristics were similar between patients with intact and whole PTH measurements (Supplementary Table S8). Interaction analysis showed that the associations between PTH levels and fracture risk were similar regardless of the PTH assay used (Supplementary Figure S3).

### Change in PTH and Subsequent Fracture Risk

**Figure 6:**
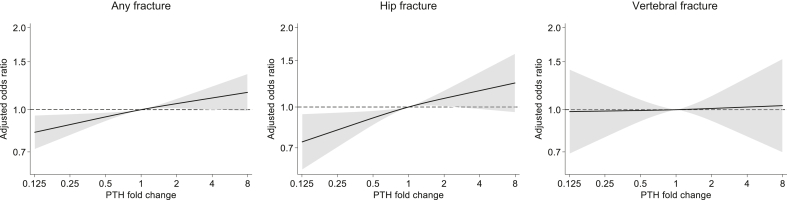
Restricted cubic splines of the adjusted odds ratio for any fracture (left), hip fracture (middle), and vertebral fracture (right) according to 1-year fold change in intact parathyroid hormone (PTH). Models adjusted for age, sex, dialysis duration, cause of kidney failure, dialysis modality (hemodialysis or hemodiafiltration), body mass index, Kt/V, normalized protein catabolic rate, history of cardiovascular disease (myocardial infarction, cerebral infarction, cerebral hemorrhage, and amputation), history of hip fracture, hemoglobin, albumin, creatinine, calcium, phosphorus, intact PTH, total cholesterol, and C-reactive protein. All covariates used for adjustment are at the time of the first intact PTH measurement. The black solid line represents the odds ratio, and the gray area represents the 95% confidence interval.

Next, we examined the percent change in intact PTH during the preceding 1 year. A total of 152,633 individuals were available for the 1-year change analysis. The median 1-year percent change in intact PTH was +1% (interquartile range, −37% to +56%). After multivariable adjustment, decreasing PTH levels over 1 year were linearly associated with a lower subsequent risk of any fracture (Table 3 and Figure 6). Consistent with the baseline model, the association with 1-year change in intact PTH was stronger for hip fracture but not found for vertebral fracture. The results were similar when we examined the 2-year change in intact PTH (Supplementary Figure S4 and Supplementary Table S9). Interaction analysis showed that the associations between the 1-year change in intact PTH and subsequent fracture risk were similar in magnitude regardless of the preceding intact PTH levels (Supplementary Figure S5). The estimated numbers-needed-to-be-exposed to >30% reduction in intact PTH to prevent 1 any fracture or 1 hip fracture over 1 year are shown in Supplementary Figure S6. The numbers-needed-to-be-exposed varied considerably between subgroups: the numbers-needed-to-be-exposed for 1 less any fracture or hip fracture were relatively low in patients aged >73 years and those with BMI of <19.9 kg/m^2^, ranging from approximately 200 to 300.

**Table 3:** Logistic regression analysis for the association between 1-year change in intact PTH and subsequent fracture

Outcome	N patients	N events	OR (95% CI) per 30% reduction in intact PTH		
			Unadjusted	Model 1	Model 2
Any fracture	152,633	3182	0.98 (0.96–0.99)	0.98 (0.97–0.99)	0.97 (0.95–0.99)
Hip fracture	152,633	1156	0.97 (0.95–1.00)	0.97 (0.95–0.99)	0.95 (0.93–0.98)
Vertebral fracture	152,633	476	0.98 (0.95–1.02)	0.99 (0.97–1.00)	1.00 (0.96–1.04)

CI, confidence interval; OR, odds ratio; PTH, parathyroid hormone.

Model 1 adjusted for age, sex, dialysis duration, cause of kidney failure, dialysis modality (hemodialysis or hemodiafiltration), body mass index, *Kt/V*, normalized protein catabolic rate, history of cardiovascular disease (myocardial infarction, cerebral infarction, cerebral hemorrhage, and amputation), and history of hip fracture.

Model 2 adjusted for Model 1 covariates plus hemoglobin, albumin, creatinine, calcium, phosphorus, intact PTH, total cholesterol, and C-reactive protein.

All covariates used for adjustment are at the time of the first intact PTH measurement.

### Fracture Risk Associated With Serum Calcium and Phosphorus

Baseline characteristics by deciles of serum calcium and phosphorus levels are shown in Supplementary Tables S10 and S11, respectively. The associations of serum calcium and phosphorus levels with fracture risk are summarized in Supplementary Tables S12 and S13, respectively, and the RCS results are shown in Supplementary Figure S7. In adjusted models, higher serum phosphorus levels were associated with an increased risk of any fracture, whereas there was no such association for serum calcium. The results were robust to sensitivity analyses (Supplementary Tables S1–S4 and S6).

## Discussion

In this analysis of a large nationwide cohort of patients on hemodialysis, we found that higher PTH levels were incrementally and approximately monotonically associated with fracture risk, with the lowest risk at the lower end of the PTH distribution. We also found that the 1-year change in PTH levels was linearly associated with subsequent fracture risk, regardless of baseline PTH levels. These associations were more pronounced for hip fractures, whereas there were no such associations for vertebral fractures. These results call for a new understanding of the potential effects of low-turnover bone on skeletal fragility and highlight the need for interventional studies to determine whether intensive PTH control reduces fracture risk in patients on hemodialysis.

Perhaps the most notable finding of this study was that the relationship between PTH levels and fracture risk was nearly linear, with the lowest PTH levels associated with the lowest risk. We also found that reductions in PTH levels over 1 year were linearly associated with a decreasing risk of subsequent fracture, even in patients with low baseline PTH levels. These results raise the possibility that aggressive lowering of PTH levels may lead to a reduction in fracture risk in patients on hemodialysis. Our results appear to contradict the traditional view that adynamic bone disease caused by PTH oversuppression leads to increased bone fragility.^5^ However, there are little bone biopsy data to support this traditional view.^12^^,^^13^ Most published data linking low PTH levels to increased fracture risk are limited to relatively small studies,^17^, ^18^, ^19^, ^20^ and no such association has been observed in larger observational studies,^14^, ^15^, ^16^ except for 1 study^21^ that did not adjust for important potential confounders such as comorbidities and nutritional status. Of note, a recent analysis of the international Dialysis Outcomes and Practice Patterns Study showed an approximately linear relationship between PTH levels and fracture risk,^32^ which is consistent with our findings. Several observational studies have also shown a linear association of fracture risk with alkaline phosphatase,^32^, ^33^, ^34^ which may better capture the extent of bone turnover associated with SHPT than PTH itself.^35^ Our results may appear to contrast with the moderate (at best) effect of cinacalcet on fracture risk in the Evaluation of Cinacalcet HCl Therapy to Lower Cardiovascular Events trial.^36^ However, a recent meta-analysis found a more substantial reduction in fracture risk with cinacalcet and newer intravenous calcimimetics, the latter of which may provide more effective PTH control through improved adherence and potentially better safety profiles.^37^ Finally, a nationwide cohort study from the United States reported a lower fracture risk in patients on hemodialysis undergoing parathyroidectomy,^38^ which typically results in postoperative PTH levels below the lower limit of the current international target range.^39^ Taken together with these previous findings, our results provide compelling evidence that low PTH levels are associated with a reduced risk of fracture and support the conduct of interventional trials to test the benefit of intensive PTH control on fracture prevention. The relationship between PTH levels and fracture risk was particularly evident for hip fracture, to which cortical rather than trabecular bone porosity contributes more.^9^ Cortical bone comprises approximately 80% of the skeleton and plays an important role in bone strength.^9^^,^^10^ This is also true for patients on hemodialysis, because bone mineral density at cortical sites has been shown to predict fracture risk in this population.^40^^,^^41^ In support of our observation, an increasing number of clinical studies have identified cortical porosity as a hallmark of hyperparathyroid bone disease.^6^, ^7^, ^8^ As a plausible mechanism for these observations, a recent experimental study has shown that elevated PTH induces osteocytic osteolysis and osteocytic cell death in cortical bone, resulting in cortical porosity.^42^ In contrast to the hip fracture results, we found no association between PTH levels and fracture risk at vertebral sites, which are largely composed of trabecular bone. Sustained elevations in PTH are known to be anabolic for trabecular bone,^6^ and it could be speculated that the anabolic effect offsets the catabolic effect of PTH on vertebral cortical bone, thus contributing to the lack of association for vertebral fracture. However, it should be noted that PTH levels were controlled at relatively low levels in this study population, which limits our ability to estimate fracture risk at much higher PTH levels. In severe SHPT, accelerated osteoclast bone resorption may exceed osteoblast bone formation, and if so, this could lead to trabecular bone loss and microarchitectural deterioration. Further studies are needed to evaluate this possibility.

The association between PTH levels and fracture risk is most likely explained by the direct effects of PTH on bone;^6^, ^7^, ^8^ however, there may be other pathways. PTH has been shown to increase energy expenditure and induce wasting,^43^ and this effect may lead to an increased risk of falls through skeletal muscle atrophy and decreased bone strength through weight loss and decreased mechanical loading. Of note, a recent report from the Dialysis Outcomes and Practice Patterns Study demonstrated a linear relationship between PTH levels and subsequent weight loss,^44^ a similar relationship pattern to that observed for fracture risk in the current study. In addition, elevated PTH has been implicated in the pathogenesis of left ventricular hypertrophy, renal anemia, and immune dysfunction.^45^ These effects can potentially lead to frailty, which in turn can increase the risk of fractures and falls.

Although the association between PTH levels and the relative risk of any fracture or hip fracture was largely consistent across subgroups, there was substantial variation in the absolute risk difference. We found that the absolute increase in fracture risk associated with higher PTH levels appeared to be more pronounced in high-risk populations, such as older individuals, females, and those with lower BMI. Consistent with our findings, the Evaluation of Cinacalcet HCl Therapy to Lower Cardiovascular Events trial reported that the effect of cinacalcet on fracture rates was more pronounced in older patients.^36^ In another study from the United States, the reduction in fracture risk associated with parathyroidectomy appeared to be more pronounced in females.^38^ For efficient and cost-effective fracture prevention, it may be reasonable to initiate or intensify PTH-lowering therapy in such patient populations. These data also raise the possibility that the high prevalence of older and underweight patients in our study may have increased our power to detect the association between PTH levels and fracture risk.

In the stratified analysis for the risk of vertebral fracture, we found a qualitative interaction by sex and serum albumin, indicating a decreased risk associated with higher PTH levels in females and in patients with higher albumin. A previous cross-sectional study reported a positive correlation between PTH levels and trabecular thickness in female patients on hemodialysis but not in males,^46^ which is consistent with the present findings. From these results, it can be speculated that the anabolic effect of PTH on vertebral trabecular bone is more pronounced in female or well-nourished patients on hemodialysis and exceeds the catabolic effect on cortical bone. Further studies are needed to investigate this possibility.

Given the deleterious effect of PTH on cortical bone,^6^, ^7^, ^8^ PTH-lowering therapy may hold promise as a means to improve cortical porosity in patients on hemodialysis. However, in a recent pilot study, treatment with etelcalcetide improved trabecular microarchitecture in the central skeleton, but there was further loss of cortical bone in the peripheral skeleton.^47^ Similarly, active vitamin D treatment was reported to increase bone mineral density in the lumbar spine but not in the cortical-rich radius.^48^ In contrast, parathyroidectomy for severe SHPT has been shown to increase bone mineral density even in the radius, although the magnitude of the increase was modest compared with the lumbar spine.^49^ These findings raise the hypothesis that when calcimimetics are used to treat SHPT, more aggressive PTH suppression, such as that seen after parathyroidectomy, may be required to improve the cortical porosity that has already occurred. It is also possible to hypothesize that early initiation of calcimimetics or active vitamin D before sustained elevation of PTH levels may be a reasonable approach to preventing the development of cortical porosity. These possibilities warrant further investigation.

The association between PTH levels and fracture risk was consistent regardless of the type of PTH assay used. Although the whole PTH assay can measure almost exclusively full-length 1 to 84 PTH, its superiority over the intact PTH assay in predicting the diagnosis of renal osteodystrophy has not been established.^50^, ^51^, ^52^ Importantly, recent studies using high-resolution mass spectrometry have confirmed the absence of circulating 7 to 84 PTH,^53^ which was previously thought to accumulate in kidney failure and exert opposite effects of 1 to 84 PTH.^54^ This finding may further challenge the rationale for preferring the whole PTH assay over the intact PTH assay. Our results provide additional evidence to support the equivalent utility of these 2 assays.

In this study, we found that higher serum phosphorus levels were associated with increased fracture risk. This finding is consistent with a recent report from a large European cohort of patients on hemodialysis.^16^ However, the mechanisms by which higher phosphorus levels may be associated with fracture risk are poorly understood. Experimental studies have shown induction of osteoblast apoptosis and inhibition of osteocyte differentiation by inorganic phosphate *in vitro*;^55^^,^^56^ however, these effects do not fully explain the observed association between serum phosphorus and fracture risk. It is also important to note that in the current study, the association between serum phosphorus levels and fracture risk was observed only for vertebral fractures, in contrast to the European cohort,^16^ which found no association for vertebral fractures. Further studies are needed to determine the effects of high phosphate levels on site-specific bone metabolism and the underlying mechanisms.

The strengths of this study include its large nationwide cohort with a substantial number of fracture events, prospective study design, fracture site information, and multiple sensitivity analyses that support the robustness of the primary results. The study has several limitations. First, this study analyzed only clinical fractures requiring hospitalization. We could only include a subset of vertebral fractures because they are often asymptomatic. We also did not include other fractures that did not require hospitalization. Therefore, we cannot exclude the possibility that the results would be different if we had data on all fracture events. Second, fracture events were recorded by local medical staff and not adjudicated by an independent committee. We were also unable to distinguish between low-energy and high-energy fractures. Third, we excluded approximately 69,000 patients from the analysis because they had missing hospitalization data for unknown reasons. Fourth, we did not have data on the manufacturers of the PTH assays used in each patient. However, all major clinical laboratories in Japan use the Elecsys intact PTH assay (Roche Diagnostics), so it is highly likely that intact PTH levels were measured by this assay in the majority of patients. Fifth, we used PTH levels measured at different times in clinical practice. Circulating PTH levels are known to have a circadian rhythm,^57^ which may be further influenced by some types of medication.^58^ This may have increased the variability of PTH levels and biased the results toward the null. Sixth, the study lacked data on the use of calcimimetics and active vitamin D. These medications may have PTH-independent effects on bone metabolism^59^^,^^60^ and may confound the association between PTH levels and fracture risk. We also lacked data on bone mineral density and bone turnover markers, which may mediate or modify the association between PTH levels and fracture risk. Seventh, the study population was limited to Japanese patients on hemodialysis, which limits the generalizability of our results. Finally, as with other observational studies, our results should not be interpreted as causal. We cannot exclude the possibility of residual confounding.

In conclusion, using data from the nationwide cohort of patients on hemodialysis, we found an approximately linear association between lower PTH levels and reduced fracture risk, particularly for hip fractures. We also found that reductions in PTH levels over 1 year were linearly associated with a decreasing risk of subsequent fractures, even in patients with low baseline PTH levels. Our results support the potential benefit of SHPT management in fracture prevention and highlight the need for randomized clinical trials to test whether intensive versus standard PTH control reduces fracture risk in patients on hemodialysis.

## Disclosure

HK has received personal fees from Chugai Pharmaceutical, Kissei Pharmaceutical, Kyowa Kirin, Ono Pharmaceutical, and Sanwa Kagaku Kenkyusho, and grants from Kyowa Kirin. TI has received personal fees and grants from Kyowa Kirin. TH has received personal fees from Chugai Pharmaceutical, Kissei Pharmaceutical, Kyowa Kirin, Ono Pharmaceutical, and Sanwa Kagaku Kenkyusho; and grants from Kissei Pharmaceutical, Kyowa Kirin, and Sanwa Kagaku Kenkyusho. NF has received personal fees from Kissei Pharmaceutical, Kyowa Kirin, Ono Pharmaceutical, and Sanwa Kagaku Kenkyusho. MA has received personal fees from Kissei Pharmaceutical, Kyowa Kirin, and Ono Pharmaceutical, and grants from Kyowa Kirin. NH has received personal fees from Chugai Pharmaceutical, Kyowa Kirin, and Ono Pharmaceutical. MF has received personal fees from Chugai Pharmaceutical, Kissei Pharmaceutical, Kyowa Kirin, Ono Pharmaceutical, and Sanwa Kagaku Kenkyusho; and grants from Chugai Pharmaceuticals, Kyowa Kirin, and Ono Pharmaceuticals.

## Supplementary Material

Supplementary File (PDF) **Figure S1.** Predicted probability of any fracture (left), hip fracture (middle), and vertebral fracture (right) across intact parathyroid hormone (PTH). **Figure S2.** Restricted cubic splines of the adjusted odds ratio for death according to intact parathyroid hormone (PTH) (left), calcium (middle), and phosphorus (right). **Figure S3.** Adjusted odds ratio for fracture associated with intact or whole parathyroid hormone (PTH). **Figure S4.** Restricted cubic splines of the adjusted odds ratio for any fracture (left), hip fracture (middle), and vertebral fracture (right) according to 2-year fold change in intact parathyroid hormone (PTH). **Figure S5.** Adjusted odds ratio for fracture associated with 1-year change in intact PTH, stratified by baseline intact parathyroid hormone (PTH). **Figure S6.** Estimated number-needed-to-be-exposed (NNE) to >30% reduction in intact parathyroid hormone (PTH) to prevent one fracture over 1 year in the overall population and relevant subgroups. **Figure S7.** Restricted cubic splines of the adjusted odds ratio for any fracture (left), hip fracture (middle), and vertebral fracture (right) according to serum calcium and phosphorus. **Table S1.** Number of missing data. **Table S2.** Poisson regression analysis for any fracture. **Table S3.** Logistic regression analysis for fracture in complete cases. **Table S4.** Logistic regression analysis for fracture using average annual measurements of biochemical parameters over 2 years. **Table S5.** Logistic regression analysis for fracture using average annual measurements of biochemical parameters over 3 years. **Table S6.** Logistic regression analysis for death. **Table S7.** Multinomial logistic regression analysis for fracture. **Table S8.** Baseline characteristics by PTH assay. **Table S9.** Logistic regression analysis for the association between 2-year change in intact PTH and subsequent fracture. **Table S10.** Baseline characteristics by deciles of serum calcium. **Table S11.** Baseline characteristics by deciles of serum phosphorus. **Table S12.** Logistic regression analysis for the association between serum calcium and fracture. **Table S13.** Logistic regression analysis for the association between serum phosphorus and fracture. **STROBE Checklist.**

## Footnotes

Supplementary File (PDF)
**Figure S1.** Predicted probability of any fracture (left), hip fracture (middle), and vertebral fracture (right) across intact parathyroid hormone (PTH). **Figure S2.** Restricted cubic splines of the adjusted odds ratio for death according to intact parathyroid hormone (PTH) (left), calcium (middle), and phosphorus (right). **Figure S3.** Adjusted odds ratio for fracture associated with intact or whole parathyroid hormone (PTH). **Figure S4.** Restricted cubic splines of the adjusted odds ratio for any fracture (left), hip fracture (middle), and vertebral fracture (right) according to 2-year fold change in intact parathyroid hormone (PTH). **Figure S5.** Adjusted odds ratio for fracture associated with 1-year change in intact PTH, stratified by baseline intact parathyroid hormone (PTH). **Figure S6.** Estimated number-needed-to-be-exposed (NNE) to >30% reduction in intact parathyroid hormone (PTH) to prevent one fracture over 1 year in the overall population and relevant subgroups. **Figure S7.** Restricted cubic splines of the adjusted odds ratio for any fracture (left), hip fracture (middle), and vertebral fracture (right) according to serum calcium and phosphorus. **Table S1.** Number of missing data. **Table S2.** Poisson regression analysis for any fracture. **Table S3.** Logistic regression analysis for fracture in complete cases. **Table S4.** Logistic regression analysis for fracture using average annual measurements of biochemical parameters over 2 years. **Table S5.** Logistic regression analysis for fracture using average annual measurements of biochemical parameters over 3 years. **Table S6.** Logistic regression analysis for death. **Table S7.** Multinomial logistic regression analysis for fracture. **Table S8.** Baseline characteristics by PTH assay. **Table S9.** Logistic regression analysis for the association between 2-year change in intact PTH and subsequent fracture. **Table S10.** Baseline characteristics by deciles of serum calcium. **Table S11.** Baseline characteristics by deciles of serum phosphorus. **Table S12.** Logistic regression analysis for the association between serum calcium and fracture. **Table S13.** Logistic regression analysis for the association between serum phosphorus and fracture.
**STROBE Checklist.**
