# Corrigendum to “Lower Parathyroid Hormone Levels are Associated With Reduced Fracture Risk in Japanese Patients on Hemodialysis” [*Kidney International Reports* Volume 9, Issue 10, October 2024, Pages 2956-2969]

**DOI:** 10.1016/j.ekir.2025.05.010

**Published:** 2025-05-14

**Authors:** Hirotaka Komaba, Takahiro Imaizumi, Takayuki Hamano, Naohiko Fujii, Masanori Abe, Norio Hanafusa, Masafumi Fukagawa

**Affiliations:** 1 Committee of Renal Data Registry, Japanese Society for Dialysis Therapy, Tokyo, Japan; 2 Division of Nephrology, Endocrinology and Metabolism, Tokai University School of Medicine, Isehara, Japan; 3 The Institute of Medical Sciences, Tokai University, Isehara, Japan; 4 Department of Advanced Medicine, Nagoya University Hospital, Nagoya, Japan; 5 Department of Nephrology, Nagoya University Graduate School of Medicine, Nagoya, Japan; 6 Department of Nephrology, Nagoya City University Graduate School of Medical Sciences, Nagoya, Japan; 7 Department of Nephrology, Osaka University Graduate School of Medicine, Suita, Japan; 8 Department of Nephrology, Hyogo Prefectural Nishinomiya Hospital, Nishinomiya, Japan; 9 Division of Nephrology, Hypertension and Endocrinology, Department of Internal Medicine, Nihon University School of Medicine, Tokyo, Japan; 10 Department of Blood Purification, Tokyo Women’s Medical University, Tokyo, Japan

The authors have requested changes to the following sentences, which remove the use of the word prospective:

Page 2957

The purpose of this study was to examine the risk of any fracture and site-specific fractures across a range of PTH levels, using data from a nationwide registry of …

Page 2957

Briefly, the JSDT sends questionnaires to all dialysis facilities in Japan at the end of each year to collect data on …

Page 2966

The strengths of this study include its large nationwide cohort with a substantial number of fracture events, fracture site information, and …

DOI of original article: https://doi.org/10.1016/j.ekir.2024.07.008

