# Supplementary material for: Lower Parathyroid Hormone Levels are Associated With Reduced Fracture Risk in Japanese Patients on Hemodialysis
Source: Kidney Int Rep. 2024 Jul 18;9(10):2956–69. doi: 10.1016/j.ekir.2024.07.008 (PMC11489479; doi:10.1016/j.ekir.2024.07.008)
Supplement: Supplementary File (PDF) — Figure S1. Predicted probability of any fracture (left), hip fracture (middle), and vertebral fracture (right) across intact parathyroid hormone (PTH). Figure S2. Restricted cubic splines of the adjusted odds ratio for death according to intact parathyroid hormone (PTH) (left), calcium (middle), and phosphorus (right). Figure S3. Adjusted odds ratio for fracture associated with intact or whole parathyroid hormone (PTH). Figure S4. Restricted cubic splines of the adjusted odds ratio for any fracture (left), hip fracture (middle), and vertebral fracture (right) according to 2-year fold change in intact parathyroid hormone (PTH). Figure S5. Adjusted odds ratio for fracture associated with 1-year change in intact PTH, stratified by baseline intact parathyroid hormone (PTH). Figure S6. Estimated number-needed-to-be-exposed (NNE) to >30% reduction in intact parathyroid hormone (PTH) to prevent one fracture over 1 year in the overall population and relevant subgroups. Figure S7. Restricted cubic splines of the adjusted odds ratio for any fracture (left), hip fracture (middle), and vertebral fracture (right) according to serum calcium and phosphorus. Table S1. Number of missing data. Table S2. Poisson regression analysis for any fracture. Table S3. Logistic regression analysis for fracture in complete cases. Table S4. Logistic regression analysis for fracture using average annual measurements of biochemical parameters over 2 years. Table S5. Logistic regression analysis for fracture using average annual measurements of biochemical parameters over 3 years. Table S6. Logistic regression analysis for death. Table S7. Multinomial logistic regression analysis for fracture. Table S8. Baseline characteristics by PTH assay. Table S9. Logistic regression analysis for the association between 2-year change in intact PTH and subsequent fracture. Table S10. Baseline characteristics by deciles of serum calcium. Table S11. Baseline characteristics by deciles of serum phosphorus. Table S12 [file mmc1.pdf]

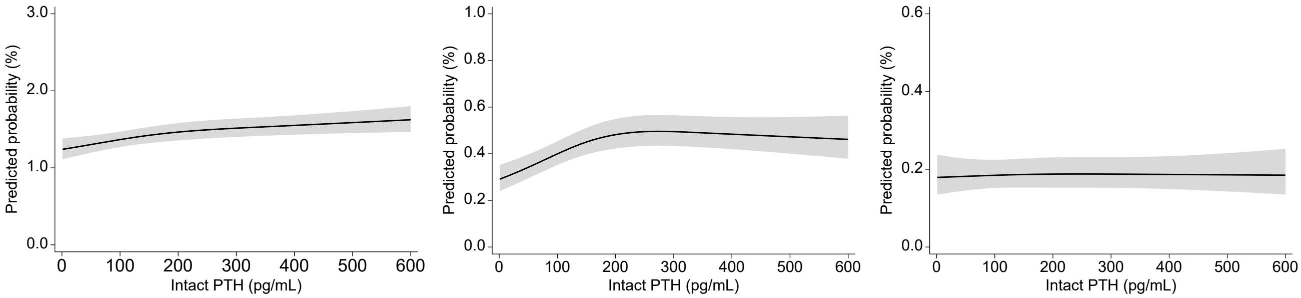


**Supplementary Figure S1.** Predicted probability of any fracture (left), hip fracture (middle), and vertebral fracture (right) across intact parathyroid hormone (PTH). Models adjusted for age, sex, dialysis duration, cause of kidney failure, dialysis modality (hemodialysis or hemodiafiltration), body mass index, *Kt/V*, normalized protein catabolic rate, history of cardiovascular disease (myocardial infarction, cerebral infarction, cerebral hemorrhage, and amputation), history of hip fracture, hemoglobin, albumin, creatinine, calcium, phosphorus, total cholesterol, and C-reactive protein. The black solid line represents the odds ratio, and the gray area represents the 95% confidence interval.

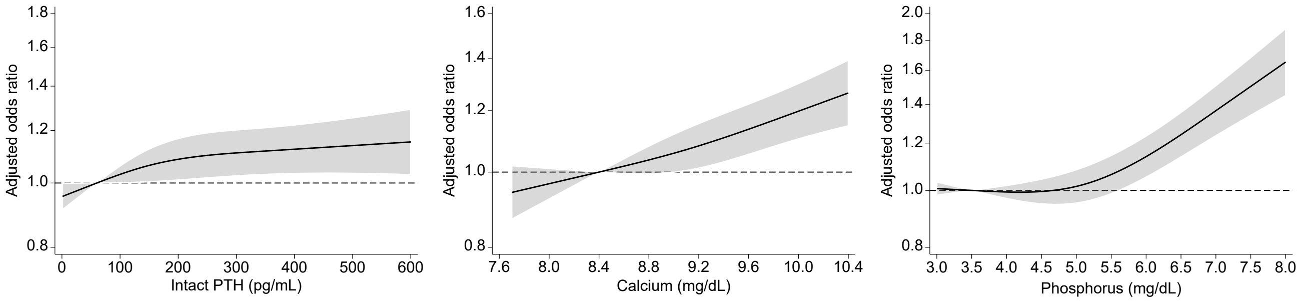


**Supplementary Figure S2.** Restricted cubic splines of the adjusted odds ratio for death according intact parathyroid hormone (PTH) (left), calcium (middle), and phosphorus (right). Models adjusted for age, sex, dialysis duration, cause of kidney failure, dialysis modality (hemodialysis or hemodiafiltration), body mass index, *Kt/V*, normalized protein catabolic rate, history of cardiovascular disease (myocardial infarction, cerebral infarction, cerebral hemorrhage, and amputation), history of hip fracture, hemoglobin, albumin, creatinine, total cholesterol, and C-reactive protein, and the 2 other predictor variables (e.g., in the PTH model, calcium and phosphorus were included as adjusting covariates). The black solid line represents the odds ratio, and the gray area represents the 95% confidence interval.

| Outcome | PTH assay | Odds ratio per doubling of PTH (95% CI) | *P* value for interaction |
|---|---|---|---|
| Any fracture | | | |
| | Intact PTH | 1.06 (1.03–1.09) | 0.57 |
| | Whole PTH | 1.08 (0.99–1.18) | |
| Hip fracture | | | |
| | Intact PTH | 1.11 (1.06–1.16) | 0.49 |
| | Whole PTH | 1.14 (0.98–1.32) | |
| Vertebral fracture | | | |
| | Intact PTH | 1.01 (0.94–1.08) | 0.72 |
| | Whole PTH | 1.05 (0.85–1.28) | |

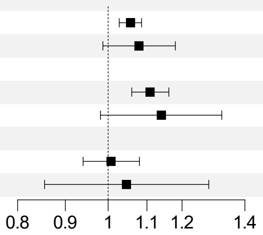


**Supplementary Figure S3.** Adjusted odds ratio for fracture associated with intact or whole parathyroid hormone (PTH). Models adjusted for age, sex, dialysis duration, cause of kidney failure, dialysis modality (hemodialysis or hemodiafiltration), body mass index, *Kt/V*, normalized protein catabolic rate, history of cardiovascular disease (myocardial infarction, cerebral infarction, cerebral hemorrhage, and amputation), history of hip fracture, hemoglobin, albumin, creatinine, calcium, phosphorus, total cholesterol, and C-reactive protein. Squares represent point estimates of the odds ratio, and horizontal lines indicate 95% confidence intervals (CIs).

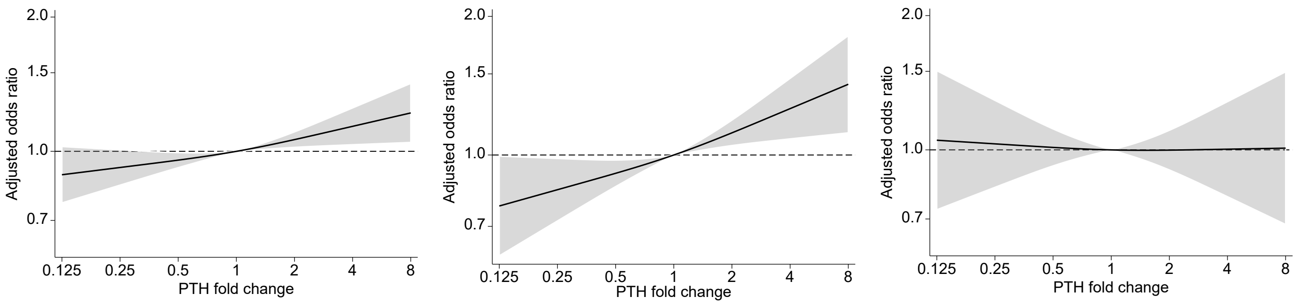


**Supplementary Figure S4.** Restricted cubic splines of the adjusted odds ratio for any fracture (left), hip fracture (middle), and vertebral fracture (right) according to 2-year fold change in intact parathyroid hormone (PTH). Models adjusted for age, sex, dialysis duration, cause of kidney failure, dialysis modality (hemodialysis or hemodiafiltration), body mass index, *Kt/V*, normalized protein catabolic rate, history of cardiovascular disease (myocardial infarction, cerebral infarction, cerebral hemorrhage, and amputation), history of hip fracture, hemoglobin, albumin, creatinine, calcium, phosphorus, intact PTH, total cholesterol, and C-reactive protein. All covariates used for adjustment are at the time of the first intact PTH measurement. The black solid line represents the odds ratio, and the gray area represents the 95% confidence interval.

| Outcome | Intact PTH, pg/mL | Odds ratio per 30% reduction in intact PTH (95% CI) | *P* value for interaction |
|---|---|---|---|
| Any fracture | | | |
| | Overall | 0.97 (0.95–0.99) | |
| | <102 | 0.98 (0.95–1.01) | 0.24 |
| | 102–196 | 0.96 (0.93–1.00) | |
| | >196 | 0.96 (0.93–0.99) | |
| Hip fracture | | | |
| | Overall | 0.95 (0.93–0.98) | |
| | <102 | 0.96 (0.92–1.00) | 0.86 |
| | 102–196 | 0.96 (0.91–1.01) | |
| | >196 | 0.95 (0.90–1.00) | |
| Vertebral fracture | | | |
| | Overall | 1.00 (0.96–1.04) | |
| | <102 | 1.02 (0.96–1.08) | 0.31 |
| | 102–196 | 0.96 (0.88–1.04) | |
| | >196 | 0.99 (0.91–1.07) | |

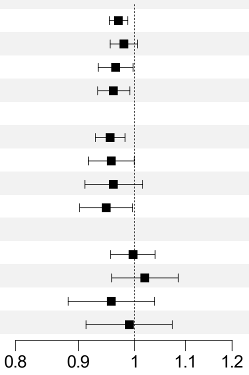


**Supplementary Figure S5.** Adjusted odds ratio for fracture associated with 1-year change in intact PTH, stratified by baseline intact parathyroid hormone (PTH). Models adjusted for age, sex, dialysis duration, cause of kidney failure, dialysis modality (hemodialysis or hemodiafiltration), body mass index, *Kt/V*, normalized protein catabolic rate, history of cardiovascular disease (myocardial infarction, cerebral infarction, cerebral hemorrhage, and amputation), history of hip fracture, hemoglobin, albumin, creatinine, calcium, phosphorus, intact PTH, total cholesterol, and C-reactive protein. All covariates used for adjustment are at the time of the first intact PTH measurement. Squares represent point estimates of the odds ratio, and horizontal lines indicate 95% confidence intervals (CIs).

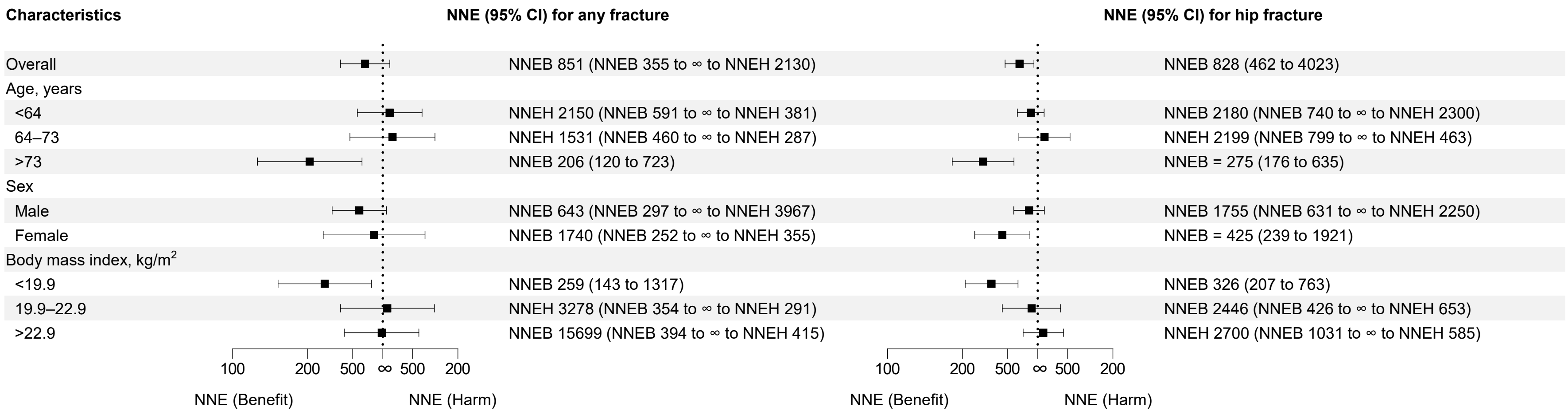


**Supplementary Figure S6.** Estimated number needed to be exposed (NNE) to >30% reduction in intact parathyroid hormone (PTH) to prevent one fracture over 1 year in the overall population and relevant subgroups. The NNE was calculated as 1/absolute risk difference for fracture associated with >30% reduction in intact PTH over 1 year, adjusted for age, sex, dialysis duration, cause of kidney failure, dialysis modality (hemodialysis or hemodiafiltration), body mass index, *Kt/V*, normalized protein catabolic rate, history of cardiovascular disease (myocardial infarction, cerebral infarction, cerebral hemorrhage, and amputation), history of hip fracture, hemoglobin, albumin, creatinine, calcium, phosphorus, intact PTH, total cholesterol, and C-reactive protein. All covariates used for adjustment are at the time of the first intact PTH measurement. Squares represent point estimates of the NNE, and horizontal lines indicate 95% confidence intervals (CIs), NNE benefit (NNEB) to ∞ to NNE harm (NNEH).

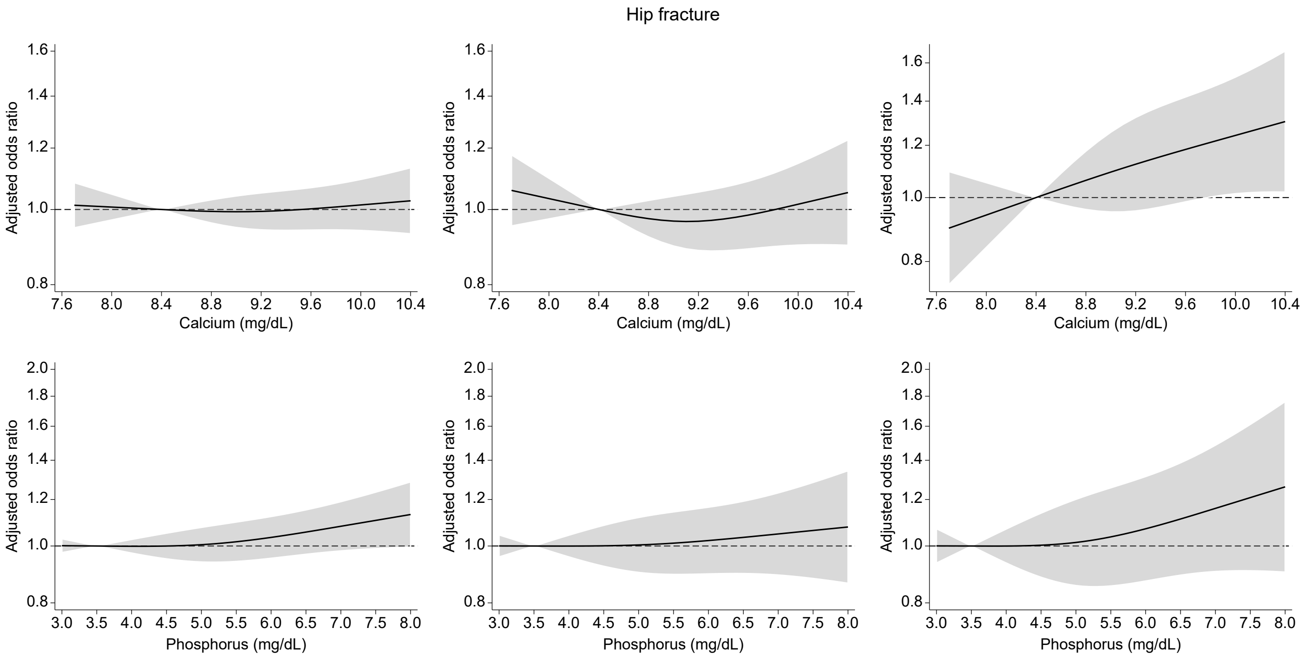


**Supplementary Figure S7.** Restricted cubic splines of the adjusted odds ratio for any fracture (left), hip fracture (middle), and vertebral fracture (right) according to serum calcium and phosphorus. Models adjusted for age, sex, dialysis duration, cause of kidney failure, dialysis modality (hemodialysis or hemodiafiltration), body mass index, *Kt/V*, normalized protein catabolic rate, history of cardiovascular disease (myocardial infarction, cerebral infarction, cerebral hemorrhage, and amputation), history of hip fracture, hemoglobin, albumin, creatinine, intact parathyroid hormone, total cholesterol, and C-reactive protein, and the other predictor variable (e.g., in the calcium model, phosphorus was included as an adjusting covariate). The black solid line represents the odds ratio, and the gray area represents the 95% confidence interval.

**Supplementary Table S1. Number of missing data**

| Characteristic | Overall (n = 180,333) |
|---|---|
| Age | 0 (0.0) |
| Female | 0 (0.0) |
| Dialysis duration | 0 (0.0) |
| Cause of kidney failure | 0 (0.0) |
| Hemodiafiltration | 0 (0.0) |
| Body mass index | 8,324 (4.6) |
| *Kt/V* | 13,936 (7.7) |
| nPCR | 13,734 (7.6) |
| Past history | |
| Myocardial infarction | 23,702 (13.1) |
| Cerebral infarction | 22,491 (12.5) |
| Cerebral hemorrhage | 24,260 (13.5) |
| Amputation | 23,644 (13.1) |
| Hip fracture | 24,114 (13.4) |
| Laboratory tests | |
| Hemoglobin | 746 (0.4) |
| Albumin | 0 (0.0) |
| Creatinine | 284 (0.2) |
| Calcium | 0 (0.0) |
| Phosphorus | 0 (0.0) |
| Intact PTH | 0 (0.0) |
| Total cholesterol | 26,989 (15.0) |
| CRP | 23,568 (13.1) |

CRP, C-reactive protein; *Kt/V*, dialysis adequacy; nPCR, normalized protein catabolic rate; PTH, parathyroid hormone.
Data are *N* (%).

**Supplementary Table S2. Poisson regression analysis for any fracture**

| Variable | | Range | *N* patients | *N* events | Adjusted RR (95% CI)[a] |
|---|---|---|---|---|---|
| Intact PTH | Per doubling | | 180,333 | 3,762 | 1.06 (1.03–1.09) |
| | Decile 1 | <39 pg/mL | 18,473 | 365 | Reference |
| | Decile 2 | 39–65 pg/mL | 17,847 | 380 | 1.12 (0.97–1.29) |
| | Decile 3 | 66–90 pg/mL | 17,812 | 384 | 1.18 (1.03–1.37) |
| | Decile 4 | 91–116 pg/mL | 18,088 | 395 | 1.22 (1.06–1.41) |
| | Decile 5 | 117–141 pg/mL | 18,183 | 398 | 1.25 (1.08–1.44) |
| | Decile 6 | 142–170 pg/mL | 18,301 | 367 | 1.17 (1.01–1.35) |
| | Decile 7 | 171–204 pg/mL | 17,611 | 369 | 1.25 (1.08–1.45) |
| | Decile 8 | 205–252 pg/mL | 17,964 | 378 | 1.28 (1.11–1.48) |
| | Decile 9 | 253–337 pg/mL | 18,046 | 370 | 1.27 (1.09–1.47) |
| | Decile 10 | >337 pg/mL | 18,008 | 356 | 1.26 (1.09–1.46) |
| Calcium | Per 1 mg/dL increase | | 180,333 | 3,762 | 1.01 (0.97–1.06) |
| | Decile 1 | <8.3 mg/dL | 15,578 | 300 | 1.04 (0.90–1.21) |
| | Decile 2 | 8.3–8.5 mg/dL | 15,894 | 328 | 1.06 (0.92–1.22) |
| | Decile 3 | 8.6–8.7 mg/dL | 19,811 | 396 | Reference |
| | Decile 4 | 8.8–8.9 mg/dL | 19,393 | 391 | 0.99 (0.86–1.13) |
| | Decile 5 | 9.0–9.1 mg/dL | 23,271 | 478 | 0.99 (0.87–1.13) |
| | Decile 6 | 9.2–9.3 mg/dL | 25,123 | 544 | 1.03 (0.90–1.17) |
| | Decile 7 | 9.4–9.5 mg/dL | 14,859 | 288 | 0.94 (0.81–1.10) |
| | Decile 8 | 9.6–9.7 mg/dL | 15,504 | 337 | 1.04 (0.90–1.20) |
| | Decile 9 | 9.8–10.0 mg/dL | 13,744 | 293 | 1.02 (0.87–1.18) |
| | Decile 10 | >10.0 mg/dL | 17,156 | 407 | 1.05 (0.91–1.22) |
| Phosphorus | Per 1 mg/dL increase | | 180,333 | 3,762 | 1.02 (1.00–1.05) |
| | Decile 1 | <3.6 mg/dL | 16,738 | 489 | 1.01 (0.88–1.15) |
| | Decile 2 | 3.6–4.0 mg/dL | 16,585 | 377 | 0.90 (0.79–1.04) |
| | Decile 3 | 4.1–4.4 mg/dL | 18,347 | 426 | Reference |
| | Decile 4 | 4.5–4.7 mg/dL | 16,188 | 349 | 0.99 (0.86–1.14) |
| | Decile 5 | 4.8–5.0 mg/dL | 17,103 | 347 | 0.96 (0.84–1.11) |
| | Decile 6 | 5.1–5.4 mg/dL | 22,389 | 486 | 1.07 (0.94–1.23) |
| | Decile 7 | 5.5–5.7 mg/dL | 15,139 | 286 | 0.98 (0.84–1.14) |
| | Decile 8 | 5.8–6.2 mg/dL | 20,411 | 354 | 0.93 (0.81–1.08) |
| | Decile 9 | 6.3–6.9 mg/dL | 18,663 | 326 | 1.01 (0.87–1.17) |
| | Decile 10 | >6.9 mg/dL | 18,770 | 322 | 1.14 (0.98–1.32) |

RR, rate ratio; PTH, parathyroid hormone.

[a]Model adjusted for age, sex, dialysis duration, cause of kidney failure, dialysis modality (hemodialysis or hemodiafiltration), body mass index, *Kt/V*, normalized protein catabolic rate, history of cardiovascular disease (myocardial infarction, cerebral infarction, cerebral hemorrhage, and amputation), history of hip fracture, hemoglobin, albumin, creatinine, total cholesterol, C-reactive protein, and the 2 other predictor variables (e.g., in the PTH model, calcium and phosphorus were included as adjusting covariates).

**Supplementary Table S3. Logistic regression analysis for fracture in complete cases**

| Variable | | Range | *N* patients | Adjusted OR (95% CI)[a] | | |
|---|---|---|---|---|---|---|
| | | | | Any fracture | Hip fracture | Vertebral fracture |
| Intact PTH | Per doubling | | 109,467 | 1.08 (1.04–1.11) | 1.12 (1.06–1.18) | 0.97 (0.90–1.05) |
| | Decile 1 | <39 pg/mL | 11,044 | Reference | Reference | Reference |
| | Decile 2 | 39–65 pg/mL | 10,794 | 1.10 (0.91–1.33) | 1.34 (0.98–1.84) | 0.88 (0.58–1.35) |
| | Decile 3 | 66–90 pg/mL | 10,908 | 1.20 (0.99–1.44) | 1.29 (0.93–1.79) | 0.96 (0.63–1.47) |
| | Decile 4 | 91–116 pg/mL | 10,977 | 1.26 (1.05–1.52) | 1.65 (1.21–2.26) | 1.15 (0.76–1.73) |
| | Decile 5 | 117–141 pg/mL | 11,036 | 1.21 (1.00–1.46) | 1.47 (1.06–2.03) | 0.80 (0.51–1.26) |
| | Decile 6 | 142–170 pg/mL | 11,223 | 1.19 (0.98–1.44) | 1.55 (1.12–2.14) | 0.96 (0.62–1.48) |
| | Decile 7 | 171–204 pg/mL | 10,692 | 1.26 (1.04–1.53) | 1.85 (1.35–2.54) | 0.79 (0.50–1.27) |
| | Decile 8 | 205–252 pg/mL | 10,909 | 1.39 (1.15–1.68) | 1.65 (1.19–2.28) | 1.16 (0.76–1.77) |
| | Decile 9 | 253–337 pg/mL | 11,025 | 1.39 (1.15–1.68) | 1.83 (1.33–2.52) | 0.89 (0.56–1.40) |
| | Decile 10 | >337 pg/mL | 10,859 | 1.29 (1.06–1.56) | 1.58 (1.14–2.21) | 0.63 (0.37–1.05) |
| Calcium | Per 1 mg/dL increase | | 109,467 | 1.04 (0.98–1.10) | 1.01 (0.91–1.12) | 1.12 (0.96–1.30) |
| | Decile 1 | <8.3 mg/dL | 9,461 | 1.00 (0.82–1.22) | 1.49 (1.07–2.08) | 0.74 (0.44–1.25) |
| | Decile 2 | 8.3–8.5 mg/dL | 9,798 | 1.10 (0.91–1.33) | 1.42 (1.02–1.96) | 1.04 (0.66–1.64) |
| | Decile 3 | 8.6–8.7 mg/dL | 12,033 | Reference | Reference | Reference |
| | Decile 4 | 8.8–8.9 mg/dL | 11,772 | 1.04 (0.87–1.25) | 1.24 (0.90–1.71) | 0.78 (0.50–1.24) |
| | Decile 5 | 9.0–9.1 mg/dL | 14,077 | 1.03 (0.86–1.22) | 1.30 (0.96–1.75) | 0.85 (0.55–1.30) |
| | Decile 6 | 9.2–9.3 mg/dL | 15,271 | 1.07 (0.91–1.27) | 1.21 (0.89–1.64) | 0.92 (0.61–1.38) |
| | Decile 7 | 9.4–9.5 mg/dL | 9,042 | 0.91 (0.74–1.11) | 1.04 (0.73–1.49) | 0.96 (0.60–1.52) |
| | Decile 8 | 9.6–9.7 mg/dL | 9,581 | 1.15 (0.95–1.39) | 1.58 (1.14–2.17) | 0.99 (0.63–1.55) |
| | Decile 9 | 9.8–10.0 mg/dL | 8,287 | 1.07 (0.87–1.30) | 1.44 (1.02–2.02) | 1.31 (0.84–2.03) |
| | Decile 10 | >10.0 mg/dL | 10,145 | 1.11 (0.92–1.34) | 1.43 (1.04–1.98) | 0.97 (0.62–1.52) |
| Phosphorus | Per 1 mg/dL increase | | 109,467 | 1.02 (0.99–1.06) | 1.03 (0.97–1.10) | 1.06 (0.97–1.16) |
| | Decile 1 | <3.6 mg/dL | 10,017 | 1.04 (0.88–1.23) | 1.04 (0.79–1.37) | 1.11 (0.74–1.68) |
| | Decile 2 | 3.6–4.0 mg/dL | 10,010 | 0.90 (0.75–1.08) | 1.07 (0.80–1.42) | 0.82 (0.52–1.29) |
| | Decile 3 | 4.1–4.4 mg/dL | 11,087 | Reference | Reference | Reference |
| | Decile 4 | 4.5–4.7 mg/dL | 9,852 | 1.00 (0.83–1.20) | 0.98 (0.72–1.34) | 0.93 (0.58–1.47) |
| | Decile 5 | 4.8–5.0 mg/dL | 10,474 | 0.90 (0.75–1.08) | 0.95 (0.69–1.29) | 0.93 (0.58–1.47) |
| | Decile 6 | 5.1–5.4 mg/dL | 13,674 | 1.08 (0.92–1.28) | 1.11 (0.83–1.47) | 1.19 (0.79–1.80) |
| | Decile 7 | 5.5–5.7 mg/dL | 9,253 | 0.91 (0.75–1.11) | 1.06 (0.77–1.47) | 1.06 (0.66–1.71) |
| | Decile 8 | 5.8–6.2 mg/dL | 12,513 | 0.87 (0.72–1.05) | 0.86 (0.62–1.18) | 1.09 (0.70–1.71) |
| | Decile 9 | 6.3–6.9 mg/dL | 11,410 | 1.03 (0.85–1.25) | 1.21 (0.89–1.66) | 0.93 (0.57–1.53) |
| | Decile 10 | >6.9 mg/dL | 11,177 | 1.20 (0.99–1.46) | 1.25 (0.89–1.75) | 1.63 (1.02–2.59) |

OR, odds ratio; PTH, parathyroid hormone.

[a]Model adjusted for age, sex, dialysis duration, cause of kidney failure, dialysis modality (hemodialysis or hemodiafiltration), body mass index, *Kt/V*, normalized protein catabolic rate, history of cardiovascular disease (myocardial infarction, cerebral infarction, cerebral hemorrhage, and amputation), history of hip fracture, hemoglobin, albumin, creatinine, total cholesterol, C-reactive protein, and the 2 other predictor variables (e.g., in the PTH model, calcium and phosphorus were included as adjusting covariates).

**Supplementary Table S4. Logistic regression analysis for fracture using average annual measurements of biochemical parameters over 2 years**

| Variable | | Range | *N* patients | Adjusted OR (95% CI)[a] | | |
|---|---|---|---|---|---|---|
| | | | | Any fracture | Hip fracture | Vertebral fracture |
| Intact PTH | Per doubling | | 152,633 | 1.04 (1.01–1.08) | 1.12 (1.06–1.18) | 0.97 (0.90–1.05) |
| | Decile 1 | <52 pg/mL | 15,255 | Reference | Reference | Reference |
| | Decile 2 | 52–80 pg/mL | 15,270 | 1.14 (0.97–1.33) | 1.55 (1.19–2.02) | 0.79 (0.60–1.04) |
| | Decile 3 | 81–105 pg/mL | 15,257 | 1.05 (0.90–1.23) | 1.36 (1.03–1.79) | 0.91 (0.64–1.30) |
| | Decile 4 | 106–128 pg/mL | 15,133 | 1.02 (0.87–1.20) | 1.29 (0.97–1.71) | 0.70 (0.47–1.04) |
| | Decile 5 | 129–150 pg/mL | 15,288 | 1.09 (0.93–1.28) | 1.55 (1.18–2.03) | 0.89 (0.74–1.08) |
| | Decile 6 | 151–175 pg/mL | 15,312 | 1.21 (1.03–1.41) | 1.72 (1.31–2.25) | 0.91 (0.61–1.35) |
| | Decile 7 | 176–204 pg/mL | 15,305 | 1.20 (1.03–1.41) | 1.54 (1.16–2.04) | 1.14 (0.95–1.37) |
| | Decile 8 | 205–244 pg/mL | 15,269 | 1.08 (0.92–1.28) | 1.72 (1.31–2.26) | 0.81 (0.55–1.20) |
| | Decile 9 | 245–315 pg/mL | 15,251 | 1.17 (1.00–1.38) | 1.67 (1.27–2.22) | 0.66 (0.44–1.00) |
| | Decile 10 | >315 pg/mL | 15,293 | 1.17 (0.99–1.37) | 1.58 (1.18–2.10) | 0.76 (0.50–1.14) |
| Calcium | Per 1 mg/dL increase | | 152,633 | 1.03 (0.97–1.09) | 1.10 (0.99–1.21) | 1.10 (0.94–1.28) |
| | Decile 1 | <8.50 mg/dL | 16,462 | 1.13 (0.97–1.32) | 1.33 (1.03–1.71) | 0.90 (0.61–1.35) |
| | Decile 2 | 8.50–8.69 mg/dL | 12,519 | 1.10 (0.93–1.29) | 1.19 (0.91–1.56) | 0.83 (0.53–1.29) |
| | Decile 3 | 8.70–8.89 mg/dL | 19,783 | Reference | Reference | Reference |
| | Decile 4 | 8.90–8.99 mg/dL | 8,360 | 0.98 (0.81–1.18) | 0.87 (0.62–1.22) | 1.08 (0.69–1.70) |
| | Decile 5 | 9.00–9.14 mg/dL | 18,719 | 1.04 (0.90–1.20) | 1.08 (0.84–1.38) | 1.06 (0.74–1.51) |
| | Decile 6 | 9.15–9.29 mg/dL | 14,597 | 1.13 (0.97–1.32) | 1.20 (0.92–1.55) | 1.05 (0.74–1.49) |
| | Decile 7 | 9.30–9.44 mg/dL | 14,273 | 1.04 (0.89–1.21) | 1.27 (0.98–1.64) | 0.98 (0.67–1.43) |
| | Decile 8 | 9.45–9.64 mg/dL | 17,864 | 1.11 (0.96–1.29) | 1.43 (1.12–1.82) | 0.84 (0.57–1.23) |
| | Decile 9 | 9.65–9.89 mg/dL | 13,872 | 1.16 (1.00–1.36) | 1.49 (1.16–1.92) | 1.08 (0.74–1.58) |
| | Decile 10 | >9.89 mg/dL | 16,184 | 1.13 (0.97–1.31) | 1.27 (0.98–1.63) | 1.21 (0.84–1.73) |
| Phosphorus | Per 1 mg/dL increase | | 152,633 | 1.06 (1.02–1.09) | 1.00 (0.94–1.07) | 1.10 (1.00–1.21) |
| | Decile 1 | <4.00 mg/dL | 16,646 | 1.14 (0.98–1.33) | 1.26 (0.98–1.60) | 1.32 (0.89–1.94) |
| | Decile 2 | 4.00–4.44 mg/dL | 17,786 | 1.11 (0.95–1.30) | 1.19 (0.92–1.52) | 0.99 (0.66–1.49) |
| | Decile 3 | 4.45–4.74 mg/dL | 14,347 | Reference | Reference | Reference |
| | Decile 4 | 4.75–4.99 mg/dL | 14,399 | 1.29 (1.10–1.52) | 1.38 (1.06–1.79) | 1.39 (0.92–2.10) |
| | Decile 5 | 5.00–5.24 mg/dL | 14,324 | 1.18 (1.00–1.39) | 1.15 (0.87–1.51) | 1.51 (1.00–2.28) |
| | Decile 6 | 5.25–5.44 mg/dL | 12,620 | 1.21 (1.02–1.44) | 1.26 (0.95–1.68) | 1.39 (0.88–2.20) |
| | Decile 7 | 5.45–5.69 mg/dL | 13,128 | 1.12 (0.94–1.33) | 1.24 (0.93–1.66) | 1.07 (0.66–1.74) |
| | Decile 8 | 5.70–6.14 mg/dL | 18,236 | 1.11 (0.94–1.31) | 1.05 (0.79–1.39) | 1.54 (1.02–2.33) |
| | Decile 9 | 6.15–6.74 mg/dL | 15,954 | 1.18 (0.99–1.40) | 1.25 (0.94–1.67) | 1.47 (0.93–2.33) |
| | Decile 10 | >6.74 mg/dL | 15,193 | 1.52 (1.27–1.81) | 1.26 (0.92–1.73) | 1.94 (1.22–3.09) |

OR, odds ratio; PTH, parathyroid hormone.

[a]Model adjusted for age, sex, dialysis duration, cause of kidney failure, dialysis modality (hemodialysis or hemodiafiltration), body mass index, *Kt/V*, normalized protein catabolic rate, history of cardiovascular disease (myocardial infarction, cerebral infarction, cerebral hemorrhage, and amputation), history of hip fracture, hemoglobin, albumin, creatinine, total cholesterol, C-reactive protein, and the 2 other predictor variables (e.g., in the PTH model, calcium and phosphorus were included as adjusting covariates).

**Supplementary Table S5. Logistic regression analysis for fracture using average annual measurements of biochemical parameters over 3 years**

| Variable | | Range | *N* patients | Adjusted OR (95% CI)[a] | | |
|---|---|---|---|---|---|---|
| | | | | Any fracture | Hip fracture | Vertebral fracture |
| Intact PTH | Per doubling | | 127,928 | 1.03 (0.99–1.07) | 1.09 (1.02–1.16) | 0.95 (0.87–1.05) |
| | Decile 1 | <57 pg/mL | 12,722 | Reference | Reference | Reference |
| | Decile 2 | 57–87 pg/mL | 12,738 | 1.13 (0.96–1.34) | 1.39 (1.06–1.84) | 0.92 (0.62–1.38) |
| | Decile 3 | 88–111 pg/mL | 12,939 | 1.00 (0.84–1.18) | 1.03 (0.76–1.39) | 0.91 (0.61–1.38) |
| | Decile 4 | 112–132 pg/mL | 12,622 | 0.99 (0.83–1.18) | 1.17 (0.87–1.58) | 0.91 (0.61–1.38) |
| | Decile 5 | 133–153 pg/mL | 12,837 | 1.14 (0.96–1.35) | 1.37 (1.02–1.83) | 0.91 (0.62–1.33) |
| | Decile 6 | 154–175 pg/mL | 12,898 | 1.10 (0.93–1.31) | 1.21 (0.90–1.63) | 1.06 (0.98–1.15) |
| | Decile 7 | 176–202 pg/mL | 12,807 | 1.18 (1.00–1.41) | 1.60 (1.20–2.13) | 1.26 (0.85–1.86) |
| | Decile 8 | 203–237 pg/mL | 12,735 | 1.12 (0.94–1.33) | 1.47 (1.10–1.97) | 0.85 (0.55–1.32) |
| | Decile 9 | 238–302 pg/mL | 12,833 | 1.10 (0.92–1.32) | 1.46 (1.08–1.96) | 0.66 (0.41–1.06) |
| | Decile 10 | >302 pg/mL | 12,797 | 1.07 (0.89–1.28) | 1.31 (0.96–1.79) | 0.64 (0.39–1.05) |
| Calcium | Per 1 mg/dL increase | | 127,928 | 1.02 (0.95–1.10) | 1.14 (1.01–1.29) | 1.07 (0.88–1.29) |
| | Decile 1 | <8.53 mg/dL | 12,694 | 1.04 (0.85–1.27) | 1.08 (0.76–1.52) | 0.97 (0.57–1.64) |
| | Decile 2 | 8.53–8.76 mg/dL | 15,453 | 1.12 (0.93–1.36) | 1.08 (0.78–1.50) | 1.11 (0.86–1.43) |
| | Decile 3 | 8.77–8.89 mg/dL | 8,552 | Reference | Reference | Reference |
| | Decile 4 | 8.90–9.02 mg/dL | 11,964 | 1.14 (0.93–1.39) | 1.10 (0.79–1.55) | 1.39 (0.87–2.23) |
| | Decile 5 | 9.03–9.16 mg/dL | 16,616 | 1.15 (0.95–1.39) | 1.10 (0.80–1.51) | 1.06 (0.66–1.71) |
| | Decile 6 | 9.17–9.29 mg/dL | 12,204 | 1.12 (0.92–1.37) | 1.25 (0.89–1.74) | 1.06 (0.64–1.77) |
| | Decile 7 | 9.30–9.42 mg/dL | 9,896 | 1.01 (0.81–1.25) | 1.02 (0.71–1.47) | 1.07 (0.66–1.74) |
| | Decile 8 | 9.43–9.59 mg/dL | 13,053 | 1.13 (0.92–1.37) | 1.26 (0.91–1.75) | 0.98 (0.60–1.59) |
| | Decile 9 | 9.60–9.86 mg/dL | 15,610 | 1.15 (0.95–1.39) | 1.23 (0.89–1.69) | 1.35 (0.84–2.18) |
| | Decile 10 | >9.86 mg/dL | 11,886 | 1.09 (0.89–1.34) | 1.39 (1.00–1.93) | 1.04 (0.62–1.75) |
| Phosphorus | Per 1 mg/dL increase | | 127,928 | 1.08 (1.03–1.13) | 1.02 (0.95–1.10) | 1.18 (1.05–1.32) |
| | Decile 1 | <4.17 mg/dL | 15,798 | 0.87 (0.75–1.02) | 0.95 (0.74–1.23) | 0.89 (0.60–1.32) |
| | Decile 2 | 4.17–4.59 mg/dL | 14,875 | 0.91 (0.77–1.07) | 0.99 (0.77–1.28) | 0.88 (0.58–1.33) |
| | Decile 3 | 4.60–4.82 mg/dL | 11,265 | Reference | Reference | Reference |
| | Decile 4 | 4.83–5.16 mg/dL | 19,713 | 0.90 (0.77–1.05) | 0.95 (0.74–1.23) | 0.87 (0.58–1.29) |
| | Decile 5 | 5.17–5.32 mg/dL | 7,292 | 0.94 (0.77–1.15) | 0.79 (0.55–1.13) | 1.30 (1.01–1.67) |
| | Decile 6 | 5.33–5.52 mg/dL | 10,469 | 0.83 (0.69–1.01) | 0.83 (0.60–1.14) | 0.95 (0.80–1.13) |
| | Decile 7 | 5.53–5.82 mg/dL | 13,662 | 0.88 (0.74–1.05) | 1.05 (0.79–1.40) | 1.20 (0.79–1.84) |
| | Decile 8 | 5.83–6.09 mg/dL | 10,104 | 0.92 (0.76–1.12) | 0.98 (0.71–1.36) | 1.05 (0.63–1.74) |
| | Decile 9 | 6.10–6.66 mg/dL | 13,311 | 1.06 (0.88–1.26) | 1.05 (0.77–1.42) | 1.62 (1.04–2.52) |
| | Decile 10 | >6.66 mg/dL | 11,439 | 1.27 (1.05–1.55) | 1.03 (0.72–1.48) | 1.63 (0.97–2.73) |

OR, odds ratio; PTH, parathyroid hormone.

[a]Model adjusted for age, sex, dialysis duration, cause of kidney failure, dialysis modality (hemodialysis or hemodiafiltration), body mass index, *Kt/V*, normalized protein catabolic rate, history of cardiovascular disease (myocardial infarction, cerebral infarction, cerebral hemorrhage, and amputation), history of hip fracture, hemoglobin, albumin, creatinine, total cholesterol, C-reactive protein, and the 2 other predictor variables (e.g., in the PTH model, calcium and phosphorus were included as adjusting covariates).

**Supplementary Table S6. Logistic regression analysis for death**

| Variable | | Range | *N* patients | *N* events | OR (95% CI) | | |
|---|---|---|---|---|---|---|---|
| | | | | | Unadjusted | Model 1 | Model 2 |
| Intact PTH | Per doubling | | 180,333 | 3,375 | 0.92 (0.90–0.95) | 0.99 (0.97–1.02) | 1.04 (1.01–1.07) |
| | Decile 1 | <39 pg/mL | 18,473 | 426 | Reference | Reference | Reference |
| | Decile 2 | 39–65 pg/mL | 17,847 | 401 | 0.97 (0.85–1.12) | 1.01 (0.88–1.16) | 1.07 (0.93–1.24) |
| | Decile 3 | 66–90 pg/mL | 17,812 | 375 | 0.91 (0.79–1.05) | 0.98 (0.85–1.13) | 1.10 (0.95–1.27) |
| | Decile 4 | 91–116 pg/mL | 18,088 | 315 | 0.75 (0.65–0.87) | 0.83 (0.71–0.96) | 0.98 (0.84–1.15) |
| | Decile 5 | 117–141 pg/mL | 18,183 | 321 | 0.76 (0.66–0.88) | 0.86 (0.74–0.99) | 1.04 (0.90–1.22) |
| | Decile 6 | 142–170 pg/mL | 18,301 | 307 | 0.72 (0.62–0.84) | 0.85 (0.73–0.99) | 1.05 (0.90–1.22) |
| | Decile 7 | 171–204 pg/mL | 17,611 | 334 | 0.82 (0.71–0.95) | 1.00 (0.86–1.16) | 1.22 (1.04–1.42) |
| | Decile 8 | 205–252 pg/mL | 17,964 | 315 | 0.76 (0.65–0.88) | 0.96 (0.83–1.11) | 1.19 (1.02–1.39) |
| | Decile 9 | 253–337 pg/mL | 18,046 | 281 | 0.67 (0.58–0.78) | 0.88 (0.75–1.03) | 1.10 (0.94–1.29) |
| | Decile 10 | >337 pg/mL | 18,008 | 300 | 0.72 (0.62–0.83) | 1.07 (0.91–1.24) | 1.23 (1.05–1.44) |
| Calcium | Per 1 mg/dL increase | | 180,333 | 3,375 | 1.47 (1.41–1.54) | 1.38 (1.32–1.44) | 1.12 (1.07–1.18) |
| | Decile 1 | <8.3 mg/dL | 15,578 | 228 | 1.13 (0.94–1.35) | 1.26 (1.05–1.51) | 1.27 (1.06–1.52) |
| | Decile 2 | 8.3–8.5 mg/dL | 15,894 | 196 | 0.95 (0.78–1.14) | 0.95 (0.79–1.15) | 0.98 (0.81–1.18) |
| | Decile 3 | 8.6–8.7 mg/dL | 19,811 | 258 | Reference | Reference | Reference |
| | Decile 4 | 8.8–8.9 mg/dL | 19,393 | 327 | 1.30 (1.10–1.53) | 1.27 (1.08–1.50) | 1.23 (1.04–1.46) |
| | Decile 5 | 9.0–9.1 mg/dL | 23,271 | 388 | 1.29 (1.10–1.51) | 1.26 (1.07–1.48) | 1.16 (0.99–1.37) |
| | Decile 6 | 9.2–9.3 mg/dL | 25,123 | 468 | 1.44 (1.23–1.68) | 1.43 (1.22–1.67) | 1.25 (1.07–1.46) |
| | Decile 7 | 9.4–9.5 mg/dL | 14,859 | 269 | 1.40 (1.18–1.66) | 1.43 (1.20–1.70) | 1.19 (1.00–1.42) |
| | Decile 8 | 9.6–9.7 mg/dL | 15,504 | 357 | 1.79 (1.52–2.10) | 1.81 (1.53–2.13) | 1.48 (1.25–1.75) |
| | Decile 9 | 9.8–10.0 mg/dL | 13,744 | 323 | 1.82 (1.55–2.15) | 1.83 (1.54–2.16) | 1.39 (1.17–1.66) |
| | Decile 10 | >10.0 mg/dL | 17,156 | 561 | 2.56 (2.21–2.97) | 2.34 (2.01–2.72) | 1.52 (1.29–1.78) |
| Phosphorus | Per 1 mg/dL increase | | 180,333 | 3,375 | 0.80 (0.78–0.82) | 0.99 (0.96–1.02) | 1.09 (1.06–1.12) |
| | Decile 1 | <3.6 mg/dL | 16,738 | 634 | 1.85 (1.63–2.11) | 1.32 (1.16–1.51) | 0.98 (0.86–1.13) |
| | Decile 2 | 3.6–4.0 mg/dL | 16,585 | 394 | 1.14 (0.99–1.32) | 1.02 (0.88–1.18) | 0.96 (0.83–1.11) |
| | Decile 3 | 4.1–4.4 mg/dL | 18,347 | 382 | Reference | Reference | Reference |
| | Decile 4 | 4.5–4.7 mg/dL | 16,188 | 274 | 0.81 (0.69–0.95) | 0.89 (0.76–1.04) | 0.94 (0.80–1.10) |
| | Decile 5 | 4.8–5.0 mg/dL | 17,103 | 293 | 0.82 (0.70–0.96) | 0.95 (0.82–1.11) | 1.06 (0.91–1.24) |
| | Decile 6 | 5.1–5.4 mg/dL | 22,389 | 349 | 0.74 (0.64–0.86) | 0.93 (0.80–1.08) | 1.06 (0.91–1.23) |
| | Decile 7 | 5.5–5.7 mg/dL | 15,139 | 216 | 0.68 (0.58–0.81) | 0.91 (0.76–1.07) | 1.05 (0.88–1.25) |
| | Decile 8 | 5.8–6.2 mg/dL | 20,411 | 278 | 0.65 (0.56–0.76) | 0.92 (0.79–1.08) | 1.08 (0.92–1.27) |
| | Decile 9 | 6.3–6.9 mg/dL | 18,663 | 276 | 0.71 (0.60–0.83) | 1.11 (0.94–1.30) | 1.30 (1.10–1.53) |
| | Decile 10 | >6.9 mg/dL | 18,770 | 279 | 0.71 (0.61–0.83) | 1.40 (1.19–1.65) | 1.57 (1.33–1.86) |

OR, odds ratio; PTH, parathyroid hormone.

Model 1 adjusted for age, sex, dialysis duration, cause of kidney failure, dialysis modality (hemodialysis or hemodiafiltration), body mass index, *Kt/V*, normalized protein catabolic rate, history of cardiovascular disease (myocardial infarction, cerebral infarction, cerebral hemorrhage, and amputation), and history of hip fracture.

Model 2 adjusted for Model 1 covariates plus hemoglobin, albumin, creatinine, total cholesterol, C-reactive protein, and the 2 other predictor variables (e.g., in the PTH model, calcium and phosphorus were included as adjusting covariates).

**Supplementary Table S7. Multinomial logistic regression analysis for fracture**

| Variable | | Range | *N* patients | Adjusted OR (95% CI)[a] | | |
|---|---|---|---|---|---|---|
| | | | | Any fracture | Hip fracture | Vertebral fracture |
| Intact PTH | Per doubling | | 180,333 | 1.04 (1.02–1.07) | 1.04 (1.01–1.07) | 1.02 (0.95–1.08) |
| | Decile 1 | <39 pg/mL | 18,473 | Reference | Reference | Reference |
| | Decile 2 | 39–65 pg/mL | 17,847 | 1.12 (0.96–1.30) | 1.30 (1.02–1.67) | 0.88 (0.60–1.27) |
| | Decile 3 | 66–90 pg/mL | 17,812 | 1.21 (1.04–1.40) | 1.26 (0.97–1.62) | 1.06 (0.76–1.49) |
| | Decile 4 | 91–116 pg/mL | 18,088 | 1.23 (1.06–1.43) | 1.38 (1.07–1.78) | 1.24 (0.89–1.72) |
| | Decile 5 | 117–141 pg/mL | 18,183 | 1.25 (1.08–1.45) | 1.37 (1.06–1.77) | 1.03 (0.72–1.47) |
| | Decile 6 | 142–170 pg/mL | 18,301 | 1.15 (0.99–1.34) | 1.59 (1.24–2.04) | 1.07 (0.75–1.53) |
| | Decile 7 | 171–204 pg/mL | 17,611 | 1.25 (1.07–1.45) | 1.90 (1.49–2.42) | 0.93 (0.63–1.39) |
| | Decile 8 | 205–252 pg/mL | 17,964 | 1.29 (1.11–1.50) | 1.65 (1.28–2.13) | 1.13 (0.78–1.62) |
| | Decile 9 | 253–337 pg/mL | 18,046 | 1.28 (1.10–1.49) | 1.67 (1.29–2.15) | 0.98 (0.67–1.45) |
| | Decile 10 | >337 pg/mL | 18,008 | 1.28 (1.10–1.50) | 1.54 (1.18–2.00) | 0.96 (0.64–1.44) |
| Calcium | Per 1 mg/dL increase | | 180,333 | 1.02 (0.97–1.07) | 1.03 (0.95–1.11) | 1.16 (1.02–1.31) |
| | Decile 1 | <8.3 mg/dL | 15,578 | 1.03 (0.88–1.21) | 1.30 (1.01–1.69) | 0.74 (0.48–1.14) |
| | Decile 2 | 8.3–8.5 mg/dL | 15,894 | 1.09 (0.94–1.27) | 1.26 (0.98–1.62) | 0.87 (0.71–1.06) |
| | Decile 3 | 8.6–8.7 mg/dL | 19,811 | Reference | Reference | Reference |
| | Decile 4 | 8.8–8.9 mg/dL | 19,393 | 0.99 (0.86–1.15) | 1.14 (0.89–1.45) | 0.94 (0.78–1.14) |
| | Decile 5 | 9.0–9.1 mg/dL | 23,271 | 0.99 (0.86–1.14) | 1.18 (0.93–1.48) | 0.92 (0.66–1.28) |
| | Decile 6 | 9.2–9.3 mg/dL | 25,123 | 1.05 (0.92–1.20) | 1.14 (0.91–1.44) | 0.90 (0.65–1.25) |
| | Decile 7 | 9.4–9.5 mg/dL | 14,859 | 0.95 (0.81–1.11) | 1.08 (0.83–1.42) | 0.94 (0.64–1.38) |
| | Decile 8 | 9.6–9.7 mg/dL | 15,504 | 1.06 (0.91–1.24) | 1.34 (1.04–1.72) | 0.99 (0.67–1.45) |
| | Decile 9 | 9.8–10.0 mg/dL | 13,744 | 1.03 (0.88–1.21) | 1.22 (0.93–1.59) | 1.30 (0.90–1.88) |
| | Decile 10 | >10.0 mg/dL | 17,156 | 1.08 (0.93–1.26) | 1.31 (1.02–1.68) | 1.11 (0.77–1.60) |
| Phosphorus | Per 1 mg/dL increase | | 180,333 | 1.03 (1.00–1.05) | 1.02 (0.97–1.06) | 1.05 (0.97–1.13) |
| | Decile 1 | <3.6 mg/dL | 16,738 | 1.02 (0.89–1.17) | 0.99 (0.80–1.23) | 1.23 (0.87–1.74) |
| | Decile 2 | 3.6–4.0 mg/dL | 16,585 | 0.90 (0.78–1.04) | 0.97 (0.77–1.21) | 1.11 (0.82–1.51) |
| | Decile 3 | 4.1–4.4 mg/dL | 18,347 | Reference | Reference | Reference |
| | Decile 4 | 4.5–4.7 mg/dL | 16,188 | 0.98 (0.85–1.14) | 0.94 (0.74–1.19) | 1.09 (0.89–1.32) |
| | Decile 5 | 4.8–5.0 mg/dL | 17,103 | 0.96 (0.83–1.11) | 0.97 (0.77–1.22) | 1.03 (0.72–1.48) |
| | Decile 6 | 5.1–5.4 mg/dL | 22,389 | 1.07 (0.93–1.22) | 0.95 (0.76–1.19) | 1.39 (1.16–1.67) |
| | Decile 7 | 5.5–5.7 mg/dL | 15,139 | 0.98 (0.84–1.14) | 0.95 (0.73–1.22) | 1.15 (0.77–1.73) |
| | Decile 8 | 5.8–6.2 mg/dL | 20,411 | 0.93 (0.80–1.07) | 0.85 (0.66–1.08) | 1.23 (0.84–1.81) |
| | Decile 9 | 6.3–6.9 mg/dL | 18,663 | 1.03 (0.88–1.19) | 1.02 (0.79–1.30) | 1.13 (0.75–1.71) |
| | Decile 10 | >6.9 mg/dL | 18,770 | 1.16 (0.99–1.36) | 1.09 (0.84–1.42) | 1.61 (1.07–2.42) |

OR, odds ratio; PTH, parathyroid hormone.

[a]Model adjusted for age, sex, dialysis duration, cause of kidney failure, dialysis modality (hemodialysis or hemodiafiltration), body mass index, *Kt/V*, normalized protein catabolic rate, history of cardiovascular disease (myocardial infarction, cerebral infarction, cerebral hemorrhage, and amputation), history of hip fracture, hemoglobin, albumin, creatinine, total cholesterol, C-reactive protein, and the 2 other predictor variables (e.g., in the PTH model, calcium and phosphorus were included as adjusting covariates).

**Supplementary Table S8. Baseline characteristics by PTH assay**

| Characteristic | Overall (n = 180,333) | PTH assay: Intact PTH (n = 165,209) | PTH assay: Whole PTH (n = 15,124) |
|---|---|---|---|
| Age, yr | 67.6 ± 12.2 | 67.6 ± 12.2 | 67.4 ± 12.3 |
| Female, % | 35.8 | 35.8 | 35.5 |
| Dialysis duration, mo | 70 (32–139) | 70 (32–138) | 74 (34–147) |
| Cause of kidney failure, % | | | |
| Glomerulonephritis | 29.7 | 29.6 | 30.7 |
| Diabetes | 38.5 | 38.6 | 37.5 |
| Hypertension | 9.8 | 9.7 | 10.3 |
| Others | 12.1 | 12.0 | 13.2 |
| Unknown | 9.9 | 10.1 | 8.4 |
| Hemodiafiltration, % | 25.1 | 25.1 | 25.2 |
| Body mass index, $kg/m^2$ | 21.9 ± 4.0 | 21.9 ± 4.0 | 21.9 ± 3.9 |
| *Kt/V* | 1.48 ± 0.31 | 1.47 ± 0.31 | 1.55 ± 0.34 |
| nPCR, g/kg/day | 0.85 ± 0.17 | 0.85 ± 0.17 | 0.86 ± 0.17 |
| Past history, % | | | |
| Myocardial infarction | 9.7 | 9.7 | 9.8 |
| Cerebral infarction | 17.1 | 17.1 | 17.8 |
| Cerebral hemorrhage | 6.2 | 6.1 | 6.5 |
| Amputation | 3.4 | 3.4 | 3.3 |
| Hip fracture | 4.5 | 4.5 | 4.4 |
| Laboratory tests | | | |
| Hemoglobin, g/dL | 10.9 ± 1.2 | 10.9 ± 1.2 | 11.0 ± 1.2 |
| Albumin, g/dL | 3.6 ± 0.4 | 3.6 ± 0.4 | 3.6 ± 0.4 |
| Creatinine, mg/dL | 10.1 ± 2.8 | 10.2 ± 2.8 | 10.0 ± 2.7 |
| Calcium, mg/dL | 9.1 ± 0.7 | 9.1 ± 0.7 | 9.2 ± 0.7 |
| Phosphorus, mg/dL | 5.2 ± 1.4 | 5.3 ± 1.4 | 5.1 ± 1.3 |
| Intact PTH, pg/mL | 141 (78–226) | 142 (79–225) | 141 (75–235) |
| Whole PTH, pg/mL | | | 83 (44–138) |
| Total cholesterol, mg/dL | 157 ± 36 | 157 ± 36 | 159 ± 35 |
| CRP, mg/dL | 0.14 (0.05–0.40) | 0.13 (0.05–0.40) | 0.14 (0.06–0.41) |

CRP, C-reactive protein; *Kt/V*, dialysis adequacy; nPCR, normalized protein catabolic rate; PTH, parathyroid hormone.
Data are percentage, mean ± SD, or median (interquartile range). Percentages do not add up to 100% in some cases because of rounding.

**Supplementary Table 9. Logistic regression analysis for the association between 2-year change in intact PTH and subsequent fracture**

| | | | OR (95% CI) per 30% reduction in intact PTH | | |
|---|---|---|---|---|---|
| Outcome | *N* patients | *N* events | Unadjusted | Model 1 | Model 2 |
| Any fracture | 132,709 | 2,802 | 0.98 (0.97–1.00) | 0.98 (0.97–1.00) | 0.97 (0.96–0.99) |
| Hip fracture | 132,709 | 1,012 | 0.97 (0.94–0.99) | 0.97 (0.95–0.99) | 0.95 (0.92–0.98) |
| Vertebral fracture | 132,709 | 418 | 0.99 (0.96–1.03) | 1.00 (0.98–1.01) | 1.00 (0.96–1.05) |

OR, odds ratio; PTH, parathyroid hormone.
Model 1 adjusted for age, sex, dialysis duration, cause of kidney failure, dialysis modality (hemodialysis or hemodiafiltration), body mass index, *Kt/V*, normalized protein catabolic rate, history of cardiovascular disease (myocardial infarction, cerebral infarction, cerebral hemorrhage, and amputation), and history of hip fracture.
Model 2 adjusted for Model 1 covariates plus hemoglobin, albumin, creatinine, calcium, phosphorus, intact PTH, total cholesterol, and C-reactive protein.
All covariates used for adjustment are at the time of the first intact PTH measurement.

**Supplementary Table S10. Baseline characteristics by deciles of serum calcium**

| | | Deciles of calcium | | | | | | | | | |
|---|---|---|---|---|---|---|---|---|---|---|---|
| Characteristic | Overall (n = 180,333) | Decile 1 <8.3 mg/dL (n = 15,578) | Decile 2 8.3–8.5 mg/dL (n = 15,894) | Decile 3 8.6–8.7 mg/dL (n = 19,811) | Decile 4 8.8–8.9 mg/dL (n = 19,393) | Decile 5 9.0–9.1 mg/dL (n = 23,271) | Decile 6 9.2–9.3 mg/dL (n = 25,123) | Decile 7 9.4–9.5 mg/dL (n = 14,859) | Decile 8 9.6–9.7 mg/dL (n = 15,504) | Decile 9 9.8–10.0 mg/dL (n = 13,744) | Decile 10 >10.0 mg/dL (n = 17,156) |
| Age, yr | 67.6 ± 12.2 | 66.3 ± 12.4 | 67.5 ± 12.5 | 67.8 ± 12.2 | 67.9 ± 12.3 | 68.0 ± 12.4 | 67.9 ± 12.2 | 67.5 ± 12.2 | 67.6 ± 12.1 | 67.3 ± 12.2 | 67.8 ± 11.9 |
| Female, % | 35.8 | 29.5 | 29.2 | 31.4 | 32.9 | 35.1 | 37.5 | 39.0 | 40.3 | 40.4 | 43.7 |
| Dialysis duration, mo | 70 (32–139) | 57 (22–129) | 55 (23–117) | 56 (24–118) | 60 (27–121) | 63 (30–124) | 71 (34–136) | 78 (37–146) | 85 (42–155) | 92 (46–163) | 103 (51–174) |
| Cause of kidney failure, % | | | | | | | | | | | |
| Glomerulonephritis | 29.7 | 27.9 | 26.3 | 26.7 | 27.0 | 28.1 | 29.4 | 31.9 | 32.5 | 34.2 | 35.7 |
| Diabetes | 38.5 | 41.5 | 43.7 | 42.1 | 41.3 | 39.9 | 38.0 | 35.7 | 34.7 | 33.2 | 32.1 |
| Hypertension | 9.8 | 9.4 | 10.0 | 10.1 | 9.9 | 10.1 | 10.1 | 9.8 | 10.0 | 9.0 | 8.6 |
| Others | 12.1 | 11.5 | 10.8 | 11.1 | 11.8 | 11.5 | 12.7 | 12.5 | 12.9 | 13.4 | 13.5 |
| Unknown | 9.9 | 9.7 | 9.1 | 10.1 | 9.9 | 10.3 | 9.8 | 10.1 | 9.8 | 10.3 | 10.1 |
| Hemodiafiltration, % | 25.1 | 25.7 | 25.3 | 24.6 | 25.0 | 24.6 | 25.3 | 25.6 | 24.9 | 26.1 | 24.7 |
| Body mass index, $kg/m^2$ | 21.9 ± 4.0 | 22.2 ± 4.1 | 22.0 ± 4.0 | 22.0 ± 4.0 | 21.9 ± 4.0 | 21.9 ± 4.0 | 21.9 ± 3.9 | 21.9 ± 4.1 | 21.8 ± 4.0 | 21.7 ± 4.0 | 21.4 ± 3.9 |
| *Kt/V* | 1.48 ± 0.31 | 1.43 ± 0.31 | 1.44 ± 0.31 | 1.46 ± 0.31 | 1.47 ± 0.31 | 1.48 ± 0.31 | 1.49 ± 0.31 | 1.50 ± 0.31 | 1.51 ± 0.31 | 1.51 ± 0.31 | 1.51 ± 0.31 |
| nPCR, g/kg/day | 0.85 ± 0.17 | 0.88 ± 0.17 | 0.86 ± 0.17 | 0.86 ± 0.17 | 0.85 ± 0.17 | 0.85 ± 0.17 | 0.85 ± 0.17 | 0.85 ± 0.17 | 0.86 ± 0.17 | 0.85 ± 0.17 | 0.84 ± 0.18 |
| Past history, % | | | | | | | | | | | |
| Myocardial infarction | 9.7 | 10.0 | 10.8 | 9.9 | 9.8 | 9.8 | 9.3 | 9.5 | 9.2 | 9.1 | 9.3 |
| Cerebral infarction | 17.1 | 15.2 | 16.4 | 16.8 | 17.4 | 17.0 | 17.5 | 16.7 | 17.3 | 17.5 | 19.4 |
| Cerebral hemorrhage | 6.2 | 5.3 | 5.3 | 5.7 | 5.6 | 6.0 | 6.3 | 6.3 | 6.4 | 6.9 | 8.2 |
| Amputation | 3.4 | 3.3 | 3.3 | 3.3 | 3.4 | 3.5 | 3.3 | 3.1 | 3.5 | 3.4 | 3.9 |
| Hip fracture | 4.5 | 4.1 | 3.8 | 3.9 | 4.2 | 4.3 | 4.6 | 4.3 | 4.8 | 5.1 | 5.7 |
| Laboratory tests | | | | | | | | | | | |
| Hemoglobin, g/dL | 10.9 ± 1.2 | 10.9 ± 1.3 | 10.9 ± 1.2 | 10.9 ± 1.2 | 10.9 ± 1.2 | 10.9 ± 1.2 | 10.9 ± 1.2 | 10.9 ± 1.2 | 10.9 ± 1.2 | 10.8 ± 1.3 | 10.8 ± 1.4 |
| Albumin, g/dL | 3.6 ± 0.4 | 3.7 ± 0.3 | 3.7 ± 0.3 | 3.7 ± 0.3 | 3.6 ± 0.4 | 3.6 ± 0.4 | 3.6 ± 0.4 | 3.6 ± 0.4 | 3.6 ± 0.4 | 3.5 ± 0.4 | 3.4 ± 0.5 |
| Creatinine, mg/dL | 10.1 ± 2.8 | 10.4 ± 2.7 | 10.2 ± 2.8 | 10.1 ± 2.8 | 10.0 ± 2.8 | 10.1 ± 2.8 | 10.1 ± 2.8 | 10.2 ± 2.8 | 10.2 ± 2.7 | 10.2 ± 2.8 | 10.0 ± 2.8 |
| Calcium, mg/dL | 9.1 ± 0.7 | 7.9 ± 0.5 | 8.4 ± 0.1 | 8.7 ± 0.1 | 8.9 ± 0.0 | 9.1 ± 0.1 | 9.3 ± 0.1 | 9.5 ± 0.0 | 9.7 ± 0.1 | 9.9 ± 0.1 | 10.5 ± 0.5 |
| Phosphorus, mg/dL | 5.2 ± 1.4 | 5.6 ± 1.6 | 5.4 ± 1.4 | 5.3 ± 1.4 | 5.2 ± 1.4 | 5.2 ± 1.3 | 5.2 ± 1.3 | 5.2 ± 1.4 | 5.2 ± 1.4 | 5.2 ± 1.4 | 5.2 ± 1.5 |
| Intact PTH, pg/mL | 141 (78–226) | 188 (111–286) | 170 (104–253) | 158 (97–238) | 150 (91–227) | 140 (83–217) | 133 (75–212) | 128 (71–209) | 122 (64–204) | 118 (60–205) | 102 (43–195) |
| Total cholesterol, mg/dL | 157 ± 36 | 156 ± 35 | 155 ± 35 | 156 ± 35 | 156 ± 35 | 157 ± 35 | 158 ± 35 | 159 ± 35 | 159 ± 36 | 158 ± 36 | 158 ± 37 |
| CRP, mg/dL | 0.14 (0.05–0.40) | 0.13 (0.05–0.38) | 0.12 (0.05–0.34) | 0.12 (0.05–0.34) | 0.13 (0.05–0.36) | 0.13 (0.05–0.38) | 0.14 (0.06–0.40) | 0.14 (0.05–0.40) | 0.14 (0.06–0.44) | 0.15 (0.06–0.48) | 0.18 (0.06–0.58) |

CRP, C-reactive protein; *Kt/V*, dialysis adequacy; nPCR, normalized protein catabolic rate; PTH, parathyroid hormone.
Data are percentage, mean ± SD, or median (interquartile range). Percentages do not add up to 100% in some cases because of rounding.

**Supplementary Table S11. Baseline characteristics by deciles of serum phosphorus**

| | | Deciles of phosphorus | | | | | | | | | |
|---|---|---|---|---|---|---|---|---|---|---|---|
| Characteristic | Overall (n = 180,333) | Decile 1 <3.6 mg/dL (n = 16,738) | Decile 2 3.6–4.0 mg/dL (n = 16,585) | Decile 3 4.1–4.4 mg/dL (n = 18,347) | Decile 4 4.5–4.7 mg/dL (n = 16,188) | Decile 5 4.8–5.0 mg/dL (n = 17,103) | Decile 6 5.1–5.4 mg/dL (n = 22,389) | Decile 7 5.5–5.7 mg/dL (n = 15,139) | Decile 8 5.8–6.2 mg/dL (n = 20,411) | Decile 9 6.3–6.9 mg/dL (n = 18,663) | Decile 10 >6.9 mg/dL (n = 18,770) |
| Age, yr | 67.6 ± 12.2 | 71.6 ± 11.5 | 70.4 ± 11.6 | 69.5 ± 11.7 | 68.9 ± 11.7 | 68.4 ± 11.7 | 67.7 ± 11.7 | 67.1 ± 11.9 | 66.3 ± 12.1 | 65.1 ± 12.4 | 62.2 ± 13.1 |
| Female, % | 35.8 | 38.1 | 36.9 | 36.8 | 36.7 | 36.0 | 36.4 | 35.9 | 35.2 | 34.3 | 32.1 |
| Dialysis duration, mo | 70 (32–139) | 66 (30–131) | 64 (29–131) | 69 (31–140) | 70 (32–142) | 71 (32–141) | 72 (33–143) | 71 (33–142) | 73 (33–142) | 73 (34–140) | 70 (33–131) |
| Cause of kidney failure, % | | | | | | | | | | | |
| Glomerulonephritis | 29.7 | 27.4 | 28.2 | 29.1 | 30.2 | 29.7 | 30.9 | 31.1 | 31.1 | 30.3 | 28.8 |
| Diabetes | 38.5 | 39.8 | 39.5 | 38.5 | 37.8 | 37.9 | 37.7 | 37.6 | 38.0 | 38.4 | 39.7 |
| Hypertension | 9.8 | 10.6 | 11.0 | 10.3 | 10.1 | 10.1 | 9.7 | 9.6 | 9.0 | 9.0 | 8.5 |
| Others | 12.1 | 11.4 | 11.3 | 12.4 | 11.9 | 12.2 | 12.1 | 12.2 | 12.2 | 12.5 | 13.0 |
| Unknown | 9.9 | 10.7 | 10.1 | 9.7 | 10.0 | 10.1 | 9.7 | 9.5 | 9.8 | 9.8 | 10.0 |
| Hemodiafiltration, % | 25.1 | 21.6 | 22.6 | 24.4 | 24.8 | 24.7 | 25.0 | 26.3 | 26.4 | 26.7 | 28.1 |
| Body mass index, kg/$m^2$ | 21.9 ± 4.0 | 20.9 ± 3.6 | 21.3 ± 3.7 | 21.5 ± 3.7 | 21.7 ± 3.8 | 21.8 ± 3.9 | 21.9 ± 3.9 | 22.1 ± 4.0 | 22.1 ± 4.1 | 22.4 ± 4.2 | 22.9 ± 4.6 |
| *Kt/V* | 1.48 ± 0.31 | 1.49 ± 0.33 | 1.50 ± 0.33 | 1.50 ± 0.31 | 1.50 ± 0.31 | 1.50 ± 0.31 | 1.49 ± 0.31 | 1.48 ± 0.30 | 1.47 ± 0.30 | 1.46 ± 0.30 | 1.43 ± 0.30 |
| nPCR, g/kg/day | 0.85 ± 0.17 | 0.74 ± 0.17 | 0.78 ± 0.16 | 0.81 ± 0.16 | 0.83 ± 0.15 | 0.84 ± 0.15 | 0.86 ± 0.15 | 0.88 ± 0.15 | 0.89 ± 0.15 | 0.92 ± 0.16 | 0.96 ± 0.17 |
| Past history, % | | | | | | | | | | | |
| Myocardial infarction | 9.7 | 10.6 | 10.2 | 9.9 | 9.7 | 9.5 | 9.7 | 9.5 | 9.5 | 9.2 | 9.1 |
| Cerebral infarction | 17.1 | 23.1 | 19.7 | 18.4 | 17.0 | 16.7 | 16.4 | 16.2 | 15.4 | 15.3 | 14.3 |
| Cerebral hemorrhage | 6.2 | 8.7 | 6.9 | 6.3 | 6.2 | 5.9 | 5.7 | 6.0 | 5.5 | 5.7 | 5.2 |
| Amputation | 3.4 | 4.8 | 3.3 | 3.2 | 3.1 | 3.4 | 3.2 | 3.0 | 3.1 | 3.3 | 3.6 |
| Hip fracture | 4.5 | 7.3 | 5.9 | 4.9 | 4.2 | 4.2 | 4.1 | 3.8 | 3.8 | 3.4 | 3.3 |
| Laboratory tests | | | | | | | | | | | |
| Hemoglobin, g/dL | 10.9 ± 1.2 | 10.5 ± 1.3 | 10.7 ± 1.2 | 10.8 ± 1.2 | 10.8 ± 1.2 | 10.9 ± 1.2 | 10.9 ± 1.2 | 10.9 ± 1.2 | 11.0 ± 1.2 | 11.0 ± 1.3 | 11.1 ± 1.4 |
| Albumin, g/dL | 3.6 ± 0.4 | 3.4 ± 0.5 | 3.5 ± 0.4 | 3.6 ± 0.4 | 3.6 ± 0.4 | 3.6 ± 0.4 | 3.6 ± 0.4 | 3.6 ± 0.4 | 3.6 ± 0.4 | 3.7 ± 0.4 | 3.7 ± 0.4 |
| Creatinine, mg/dL | 10.1 ± 2.8 | 8.3 ± 2.8 | 9.1 ± 2.7 | 9.5 ± 2.7 | 9.8 ± 2.6 | 10.0 ± 2.6 | 10.3 ± 2.5 | 10.5 ± 2.5 | 10.8 ± 2.5 | 11.1 ± 2.6 | 11.7 ± 2.7 |
| Calcium, mg/dL | 9.1 ± 0.7 | 9.3 ± 0.8 | 9.2 ± 0.7 | 9.2 ± 0.7 | 9.2 ± 0.7 | 9.1 ± 0.7 | 9.1 ± 0.7 | 9.1 ± 0.7 | 9.1 ± 0.7 | 9.1 ± 0.7 | 9.1 ± 0.8 |
| Phosphorus, mg/dL | 5.2 ± 1.4 | 3.0 ± 0.5 | 3.8 ± 0.1 | 4.3 ± 0.1 | 4.6 ± 0.1 | 4.9 ± 0.1 | 5.2 ± 0.1 | 5.6 ± 0.1 | 6.0 ± 0.1 | 6.6 ± 0.2 | 7.9 ± 1.0 |
| Intact PTH, pg/mL | 141 (78–226) | 103 (54–171) | 122 (67–194) | 129 (73–204) | 137 (77–213) | 138 (79–217) | 146 (83–229) | 150 (85–234) | 154 (87–241) | 162 (92–256) | 183 (100–295) |
| Total cholesterol, mg/dL | 157 ± 36 | 150 ± 36 | 153 ± 35 | 155 ± 35 | 156 ± 35 | 157 ± 35 | 158 ± 35 | 158 ± 35 | 159 ± 36 | 160 ± 36 | 162 ± 37 |
| CRP, mg/dL | 0.14 (0.05–0.40) | 0.18 (0.06–0.64) | 0.14 (0.05–0.45) | 0.13 (0.05–0.40) | 0.13 (0.05–0.37) | 0.12 (0.05–0.35) | 0.12 (0.05–0.34) | 0.12 (0.05–0.36) | 0.12 (0.05–0.35) | 0.14 (0.06–0.38) | 0.18 (0.07–0.48) |

CRP, C-reactive protein; *Kt/V*, dialysis adequacy; nPCR, normalized protein catabolic rate; PTH, parathyroid hormone.
Data are percentage, mean ± SD, or median (interquartile range). Percentages do not add up to 100% in some cases because of rounding.

**Supplementary Table S12. Logistic regression analysis for the association between serum calcium and fracture**

| | | | | | OR (95% CI) | | |
|---|---|---|---|---|---|---|---|
| Outcome | Calcium | Range | *N* patients | *N* events | Unadjusted | Model 1 | Model 2 |
| Any fracture | Per 1 mg/dL increase | | 180,333 | 3,708 | 1.08 (1.03–1.13) | 1.01 (0.97–1.06) | 1.01 (0.97–1.06) |
| | Decile 1 | <8.3 mg/dL | 15,578 | 291 | 0.94 (0.81–1.10) | 1.02 (0.88–1.20) | 1.02 (0.87–1.19) |
| | Decile 2 | 8.3–8.5 mg/dL | 15,894 | 328 | 1.04 (0.90–1.21) | 1.07 (0.92–1.24) | 1.07 (0.92–1.24) |
| | Decile 3 | 8.6–8.7 mg/dL | 19,811 | 393 | Reference | Reference | Reference |
| | Decile 4 | 8.8–8.9 mg/dL | 19,393 | 385 | 1.00 (0.87–1.15) | 0.98 (0.85–1.13) | 0.98 (0.85–1.13) |
| | Decile 5 | 9.0–9.1 mg/dL | 23,271 | 473 | 1.03 (0.90–1.17) | 0.99 (0.86–1.13) | 0.99 (0.86–1.13) |
| | Decile 6 | 9.2–9.3 mg/dL | 25,123 | 537 | 1.08 (0.95–1.23) | 1.02 (0.90–1.17) | 1.03 (0.90–1.17) |
| | Decile 7 | 9.4–9.5 mg/dL | 14,859 | 281 | 0.95 (0.82–1.11) | 0.92 (0.79–1.08) | 0.93 (0.79–1.08) |
| | Decile 8 | 9.6–9.7 mg/dL | 15,504 | 332 | 1.08 (0.93–1.25) | 1.03 (0.88–1.19) | 1.03 (0.89–1.20) |
| | Decile 9 | 9.8–10.0 mg/dL | 13,744 | 287 | 1.05 (0.90–1.23) | 1.00 (0.86–1.17) | 1.01 (0.86–1.18) |
| | Decile 10 | >10.0 mg/dL | 17,156 | 401 | 1.18 (1.03–1.36) | 1.06 (0.92–1.22) | 1.05 (0.91–1.22) |
| Hip fracture | Per 1 mg/dL increase | | 180,333 | 1,361 | 1.08 (1.00–1.16) | 0.98 (0.91–1.06) | 1.02 (0.94–1.11) |
| | Decile 1 | <8.3 mg/dL | 15,578 | 116 | 1.17 (0.91–1.51) | 1.32 (1.02–1.70) | 1.30 (1.00–1.67) |
| | Decile 2 | 8.3–8.5 mg/dL | 15,894 | 124 | 1.23 (0.96–1.58) | 1.28 (0.99–1.64) | 1.26 (0.98–1.62) |
| | Decile 3 | 8.6–8.7 mg/dL | 19,811 | 126 | Reference | Reference | Reference |
| | Decile 4 | 8.8–8.9 mg/dL | 19,393 | 142 | 1.15 (0.91–1.47) | 1.13 (0.88–1.43) | 1.13 (0.89–1.44) |
| | Decile 5 | 9.0–9.1 mg/dL | 23,271 | 178 | 1.20 (0.96–1.51) | 1.15 (0.91–1.45) | 1.17 (0.93–1.48) |
| | Decile 6 | 9.2–9.3 mg/dL | 25,123 | 188 | 1.18 (0.94–1.48) | 1.10 (0.88–1.38) | 1.14 (0.90–1.43) |
| | Decile 7 | 9.4–9.5 mg/dL | 14,859 | 101 | 1.07 (0.82–1.39) | 1.03 (0.79–1.35) | 1.08 (0.83–1.41) |
| | Decile 8 | 9.6–9.7 mg/dL | 15,504 | 130 | 1.32 (1.03–1.69) | 1.24 (0.97–1.59) | 1.32 (1.02–1.69) |
| | Decile 9 | 9.8–10.0 mg/dL | 13,744 | 105 | 1.20 (0.93–1.56) | 1.12 (0.86–1.46) | 1.20 (0.92–1.56) |
| | Decile 10 | >10.0 mg/dL | 17,156 | 151 | 1.39 (1.09–1.76) | 1.18 (0.93–1.50) | 1.28 (1.00–1.65) |
| Vertebral fracture | Per 1 mg/dL increase | | 180,333 | 551 | 1.27 (1.14–1.43) | 1.01 (0.97–1.06) | 1.15 (1.02–1.30) |
| | Decile 1 | <8.3 mg/dL | 15,578 | 30 | 0.64 (0.41–0.99) | 0.73 (0.58–0.91) | 0.74 (0.71–0.77) |
| | Decile 2 | 8.3–8.5 mg/dL | 15,894 | 40 | 0.83 (0.56–1.24) | 0.85 (0.70–1.05) | 0.87 (0.71–1.06) |
| | Decile 3 | 8.6–8.7 mg/dL | 19,811 | 60 | Reference | Reference | Reference |
| | Decile 4 | 8.8–8.9 mg/dL | 19,393 | 57 | 0.97 (0.67–1.40) | 0.95 (0.79–1.14) | 0.94 (0.78–1.13) |
| | Decile 5 | 9.0–9.1 mg/dL | 23,271 | 69 | 0.98 (0.69–1.38) | 0.94 (0.69–1.28) | 0.92 (0.65–1.28) |
| | Decile 6 | 9.2–9.3 mg/dL | 25,123 | 74 | 0.97 (0.69–1.37) | 0.93 (0.76–1.13) | 0.90 (0.87–0.92) |
| | Decile 7 | 9.4–9.5 mg/dL | 14,859 | 45 | 1.00 (0.68–1.47) | 0.98 (0.68–1.41) | 0.94 (0.65–1.36) |
| | Decile 8 | 9.6–9.7 mg/dL | 15,504 | 50 | 1.07 (0.73–1.55) | 1.03 (0.71–1.48) | 0.98 (0.67–1.43) |
| | Decile 9 | 9.8–10.0 mg/dL | 13,744 | 59 | 1.42 (0.99–2.03) | 1.38 (0.96–1.98) | 1.29 (0.89–1.87) |
| | Decile 10 | >10.0 mg/dL | 17,156 | 67 | 1.29 (0.91–1.83) | 1.22 (0.86–1.73) | 1.09 (0.76–1.57) |

OR, odds ratio; PTH, parathyroid hormone.

Model 1 adjusted for age, sex, dialysis duration, cause of kidney failure, dialysis modality (hemodialysis or hemodiafiltration), body mass index, *Kt/V*, normalized protein catabolic rate, history of cardiovascular disease (myocardial infarction, cerebral infarction, cerebral hemorrhage, and amputation), and history of hip fracture.

Model 2 adjusted for Model 1 covariates plus hemoglobin, albumin, creatinine, phosphorus, intact PTH, total cholesterol, and C-reactive protein

**Supplementary Table S13. Logistic regression analysis for the association between serum phosphorus and fracture**

| Outcome | Phosphorus | Range | *N* patients | *N* events | OR (95% CI) | | |
|---|---|---|---|---|---|---|---|
| | | | | | Unadjusted | Model 1 | Model 2 |
| Any fracture | Per 1 mg/dL increase | | 180,333 | 3,708 | 0.89 (0.87–0.92) | 1.01 (0.99–1.04) | 1.02 (1.00–1.05) |
| | Decile 1 | <3.6 mg/dL | 16,738 | 484 | 1.27 (1.11–1.45) | 1.05 (0.92–1.20) | 1.02 (0.89–1.17) |
| | Decile 2 | 3.6–4.0 mg/dL | 16,585 | 370 | 0.97 (0.85–1.12) | 0.90 (0.78–1.04) | 0.90 (0.78–1.03) |
| | Decile 3 | 4.1–4.4 mg/dL | 18,347 | 420 | Reference | Reference | Reference |
| | Decile 4 | 4.5–4.7 mg/dL | 16,188 | 345 | 0.93 (0.80–1.07) | 0.98 (0.85–1.14) | 0.99 (0.86–1.14) |
| | Decile 5 | 4.8–5.0 mg/dL | 17,103 | 344 | 0.88 (0.76–1.01) | 0.95 (0.83–1.10) | 0.97 (0.84–1.12) |
| | Decile 6 | 5.1–5.4 mg/dL | 22,389 | 473 | 0.92 (0.81–1.05) | 1.04 (0.91–1.20) | 1.06 (0.93–1.21) |
| | Decile 7 | 5.5–5.7 mg/dL | 15,139 | 281 | 0.81 (0.69–0.94) | 0.95 (0.82–1.11) | 0.97 (0.83–1.14) |
| | Decile 8 | 5.8–6.2 mg/dL | 20,411 | 349 | 0.74 (0.64–0.86) | 0.91 (0.79–1.05) | 0.93 (0.80–1.08) |
| | Decile 9 | 6.3–6.9 mg/dL | 18,663 | 323 | 0.75 (0.65–0.87) | 0.99 (0.85–1.15) | 1.01 (0.87–1.18) |
| | Decile 10 | >6.9 mg/dL | 18,770 | 319 | 0.74 (0.64–0.85) | 1.13 (0.97–1.31) | 1.14 (0.98–1.33) |
| Hip fracture | Per 1 mg/dL increase | | 180,333 | 1,361 | 0.84 (0.81–0.88) | 1.01 (0.96–1.05) | 1.01 (0.97–1.06) |
| | Decile 1 | <3.6 mg/dL | 16,738 | 198 | 1.33 (1.08–1.63) | 0.98 (0.80–1.22) | 0.98 (0.79–1.22) |
| | Decile 2 | 3.6–4.0 mg/dL | 16,585 | 160 | 1.08 (0.87–1.34) | 0.97 (0.78–1.21) | 0.97 (0.78–1.21) |
| | Decile 3 | 4.1–4.4 mg/dL | 18,347 | 164 | Reference | Reference | Reference |
| | Decile 4 | 4.5–4.7 mg/dL | 16,188 | 125 | 0.86 (0.68–1.09) | 0.94 (0.74–1.19) | 0.95 (0.75–1.20) |
| | Decile 5 | 4.8–5.0 mg/dL | 17,103 | 129 | 0.84 (0.67–1.06) | 0.95 (0.75–1.20) | 0.97 (0.77–1.22) |
| | Decile 6 | 5.1–5.4 mg/dL | 22,389 | 158 | 0.79 (0.63–0.98) | 0.94 (0.75–1.17) | 0.95 (0.76–1.19) |
| | Decile 7 | 5.5–5.7 mg/dL | 15,139 | 99 | 0.73 (0.57–0.94) | 0.93 (0.72–1.19) | 0.95 (0.73–1.22) |
| | Decile 8 | 5.8–6.2 mg/dL | 20,411 | 115 | 0.63 (0.49–0.80) | 0.83 (0.65–1.06) | 0.85 (0.66–1.08) |
| | Decile 9 | 6.3–6.9 mg/dL | 18,663 | 112 | 0.67 (0.53–0.85) | 0.98 (0.77–1.26) | 1.01 (0.79–1.29) |
| | Decile 10 | >6.9 mg/dL | 18,770 | 101 | 0.60 (0.47–0.77) | 1.06 (0.82–1.37) | 1.08 (0.83–1.41) |
| Vertebral fracture | Per 1 mg/dL increase | | 180,333 | 551 | 0.86 (0.81–0.92) | 1.01 (0.99–1.04) | 1.04 (0.97–1.12) |
| | Decile 1 | <3.6 mg/dL | 16,738 | 82 | 1.64 (1.16–2.31) | 1.33 (0.94–1.88) | 1.23 (0.86–1.74) |
| | Decile 2 | 3.6–4.0 mg/dL | 16,585 | 61 | 1.23 (0.85–1.77) | 1.13 (0.81–1.60) | 1.11 (0.80–1.55) |
| | Decile 3 | 4.1–4.4 mg/dL | 18,347 | 55 | Reference | Reference | Reference |
| | Decile 4 | 4.5–4.7 mg/dL | 16,188 | 49 | 1.01 (0.69–1.48) | 1.08 (0.89–1.32) | 1.09 (0.89–1.33) |
| | Decile 5 | 4.8–5.0 mg/dL | 17,103 | 46 | 0.90 (0.61–1.33) | 1.01 (0.70–1.46) | 1.03 (0.71–1.51) |
| | Decile 6 | 5.1–5.4 mg/dL | 22,389 | 76 | 1.13 (0.80–1.60) | 1.36 (1.14–1.62) | 1.39 (1.16–1.67) |
| | Decile 7 | 5.5–5.7 mg/dL | 15,139 | 40 | 0.88 (0.59–1.32) | 1.11 (0.90–1.38) | 1.15 (0.76–1.73) |
| | Decile 8 | 5.8–6.2 mg/dL | 20,411 | 54 | 0.88 (0.61–1.28) | 1.20 (0.86–1.66) | 1.23 (0.84–1.81) |
| | Decile 9 | 6.3–6.9 mg/dL | 18,663 | 41 | 0.73 (0.49–1.10) | 1.09 (0.76–1.57) | 1.13 (0.75–1.69) |
| | Decile 10 | >6.9 mg/dL | 18,770 | 47 | 0.83 (0.57–1.23) | 1.55 (1.04–2.32) | 1.59 (1.05–2.39) |

OR, odds ratio; PTH, parathyroid hormone.

Model 1 adjusted for age, sex, dialysis duration, cause of kidney failure, dialysis modality (hemodialysis or hemodiafiltration), body mass index, *Kt/V*, normalized protein catabolic rate, history of cardiovascular disease (myocardial infarction, cerebral infarction, cerebral hemorrhage, and amputation), and history of hip fracture.

Model 2 adjusted for Model 1 covariates plus hemoglobin, albumin, creatinine, calcium, intact PTH, total cholesterol, and C-reactive protein.

STROBE Statement—Checklist of items that should be included in reports of ***cohort studies***

| | Item No | Recommendation | Page No |
|---|---|---|---|
| **Title and abstract** | 1 | (*a*) Indicate the study's design with a commonly used term in the title or the abstract<br>(*b*) Provide in the abstract an informative and balanced summary of what was done and what was found | 1-2 |
| **Introduction** | | | |
| Background/rationale | 2 | Explain the scientific background and rationale for the investigation being reported | 3-4 |
| Objectives | 3 | State specific objectives, including any prespecified hypotheses | 3-4 |
| **Methods** | | | |
| Study design | 4 | Present key elements of study design early in the paper | 4-6 |
| Setting | 5 | Describe the setting, locations, and relevant dates, including periods of recruitment, exposure, follow-up, and data collection | 4-6 |
| Participants | 6 | (*a*) Give the eligibility criteria, and the sources and methods of selection of participants. Describe methods of follow-up<br>(*b*) For matched studies, give matching criteria and number of exposed and unexposed | 4-6 |
| Variables | 7 | Clearly define all outcomes, exposures, predictors, potential confounders, and effect modifiers. Give diagnostic criteria, if applicable | 6-8 |
| Data sources/ measurement | 8* | For each variable of interest, give sources of data and details of methods of assessment (measurement). Describe comparability of assessment methods if there is more than one group | 4-6 |
| Bias | 9 | Describe any efforts to address potential sources of bias | 6-8 |
| Study size | 10 | Explain how the study size was arrived at | 4-6 |
| Quantitative variables | 11 | Explain how quantitative variables were handled in the analyses. If applicable, describe which groupings were chosen and why | 6-8 |
| Statistical methods | 12 | (*a*) Describe all statistical methods, including those used to control for confounding<br>(*b*) Describe any methods used to examine subgroups and interactions<br>(*c*) Explain how missing data were addressed<br>(*d*) If applicable, explain how loss to follow-up was addressed<br>(*e*) Describe any sensitivity analyses | 6-8 |
| **Results** | | | |
| Participants | 13* | (a) Report numbers of individuals at each stage of study—eg numbers potentially eligible, examined for eligibility, confirmed eligible, included in the study, completing follow-up, and analysed<br>(b) Give reasons for non-participation at each stage<br>(c) Consider use of a flow diagram | 8 |
| Descriptive data | 14* | (a) Give characteristics of study participants (eg demographic, clinical, social) and information on exposures and potential confounders | 8 |

| | | | |
|---|---|---|---|
| | | (b) Indicate number of participants with missing data for each variable of interest<br>(c) Summarise follow-up time (eg, average and total amount) | |
| Outcome data | 15* | Report numbers of outcome events or summary measures over time | 9 |
| Main results | 16 | (*a*) Give unadjusted estimates and, if applicable, confounder-adjusted estimates and their precision (eg, 95% confidence interval). Make clear which confounders were adjusted for and why they were included<br>(*b*) Report category boundaries when continuous variables were categorized<br>(*c*) If relevant, consider translating estimates of relative risk into absolute risk for a meaningful time period | 9-11 |
| Other analyses | 17 | Report other analyses done—eg analyses of subgroups and interactions, and sensitivity analyses | 9-11 |
| **Discussion** | | | |
| Key results | 18 | Summarise key results with reference to study objectives | 11-12 |
| Limitations | 19 | Discuss limitations of the study, taking into account sources of potential bias or imprecision. Discuss both direction and magnitude of any potential bias | 16-17 |
| Interpretation | 20 | Give a cautious overall interpretation of results considering objectives, limitations, multiplicity of analyses, results from similar studies, and other relevant evidence | 11-18 |
| Generalisability | 21 | Discuss the generalisability (external validity) of the study results | 17 |
| **Other information** | | | |
| Funding | 22 | Give the source of funding and the role of the funders for the present study and, if applicable, for the original study on which the present article is based | 18 |

*Give information separately for exposed and unexposed groups.

**Note:** An Explanation and Elaboration article discusses each checklist item and gives methodological background and published examples of transparent reporting. The STROBE checklist is best used in conjunction with this article (freely available on the Web sites of PLoS Medicine at http://www.plosmedicine.org/, Annals of Internal Medicine at http://www.annals.org/, and Epidemiology at http://www.epidem.com/). Information on the STROBE Initiative is available at http://www.strobe-statement.org.
